# Giant Photoluminescence Enhancement of Ga‐Doped ZnO Microwires by X‐Ray Irradiation

**DOI:** 10.1002/advs.202407144

**Published:** 2024-11-25

**Authors:** Siyuan He, Shuiyan Cao, Ying Liu, Wenfa Chen, Pin Lyu, Weidian Li, Jincheng Bao, Wenhui Sun, Caixia Kan, Mingming Jiang, Yanpeng Liu

**Affiliations:** ^1^ College of Physics Key Laboratory for Intelligent Nano Materials and Devices of Ministry of Education Nanjing University of Aeronautics and Astronautics Nanjing 210016 China; ^2^ State Key Laboratory of Mechanics and Control of Mechanical Structures and Institute for Frontier Science Nanjing University of Aeronautics and Astronautics Nanjing 210016 China; ^3^ Key Laboratory of Aerospace Information Materials and Physics Ministry of Industry and Information Technology Nanjing 210016 China; ^4^ National Key Laboratory of Microwave Photonics Nanjing University of Aeronautics and Astronautics Nanjing 210016 China

**Keywords:** lattice relaxation, oxygen vacancy, photoluminescence, X‐ray irradiation, ZnO microwire

## Abstract

Ga‐doped zinc oxide (ZnO) microwires hold great promise for developing highly efficient light sources because of the wide bandgap with proper exciton binding energy. However, most microwires grown from one mainstream approach, i.e., chemical vapor deposition (CVD), are morphologically and crystallographically defective, exhibiting limited photoluminescence performances. Herein, a simple and effective X‐ray irradiation strategy is demonstrated for enhancing the photoluminescence of Ga‐doped ZnO microwire in ambient conditions. Under moderate doses (≤ 150 Gy), the photoluminescence monotonically rockets up with X‐ray dose increment and achieves nine‐fold enhancement at a dose of ≈150 Gy, recording high photoluminescence improvement of ZnO microwires to date. The elemental characteristics under different controlled irradiation atmospheres suggest the elimination of surface oxygen vacancy and the cross‐section transmission electron microscope reveals prominent lattice relaxations after mild X‐ray irradiation. In addition, the X‐ray irradiated microwires further exhibit elevated electroluminescence by over three times. The enhanced photoluminescence and electroluminescence as well as long‐term stability enable us to imagine the super‐rapid applications of ZnO microwires in modern optoelectronic devices.

## Graphical abstract

A straightforward and effective X‐ray irradiation strategy enhances the photoluminescence of Ga‐doped ZnO microwires under ambient conditions, achieving a nine‐fold increment at a dose of about 150 Gy. This enhancement results from the reduction of surface oxygen vacancies and lattice relaxation. Furthermore, this method demonstrates promising potentials for applications in the field of electroluminescence.

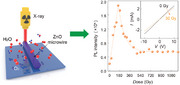

## 1 Introduction

ZnO microwires, due to their direct bandgap of ≈3.37 eV, high exciton binding energy of 60 meV, and non‐toxicity,^[^ ^1^ ^]^ are widely adopted in tremendous optoelectronic applications, including ultraviolet lasers, photodetectors, solar cells, ultraviolet light‐emitting diodes, and catalysts.^[^ ^2^, ^3^, ^4^, ^5^, ^6^, ^7^, ^8^, ^9^ ^]^ Prompted by the huge demand for ZnO‐based devices with superior performances, the photoluminescence (PL) of ZnO crystals, one key figure of merit for optoelectronics, attracts intense fundamental investigations from various defect engineering, doping, and other strengthening approaches. Despite diverse bottom‐up synthesis protocols, the fresh ZnO microwires avoidably consist of oxygen vacancies, lattice distortions, structural defects, and elemental impurities, which slightly split the PL peak into pristine exciton one (≈380 nm) and additional deep level one. To solve these defective issues, several approaches including polymer covering,^[^ ^10^ ^]^ argon ion milling,^[^ ^11^ ^]^ and surface chemical passivation^[^ ^12^, ^13^ ^]^ were reported in succession with the trade‐off of increased surface roughness and curvatures.

Recently, the incorporations of certain metal dopants into ZnO nano/microstructures, such as Al, and Li, effectively promote the PL and ultraviolet emissions by ≤3 times.^[^ ^14^, ^15^, ^16^, ^17^, ^18^, ^19^, ^20^ ^]^ Among these, the Ga atom shares a comparable radius with the Zn atom. The introduction of Ga atoms significantly enhances the ultraviolet emission and redshifts the electroluminescence of ZnO nanostructures. At finite doping concentrations, the foreign Ga atoms into the host ZnO lattice lead to negligible ZnO lattice distortions and other structural defects.^[^ ^21^, ^22^, ^23^ ^]^ In the frame of the Burstein‐Moss effect, the implantations of Ga atoms progressively blue‐shift the near‐band edge emission of host ZnO structures from ≈380 to ≈370 nm.^[^ ^24^ ^]^ Departing from this, one metallic nanoparticle capping over Ga‐doped ZnO crystals^[^ ^25^, ^26^, ^27^, ^28^ ^]^ and high‐temperature annealing show great potentials in elevating the near‐band edge emission, but with the aggravations of morphological and structural degradations.^[^ ^29^, ^30^ ^]^ In early attempts, we also employed various noble metal nanoparticles with matchable resonance absorption energy to enhance the PL emission of ZnO microwires.^[^ ^26^, ^31^, ^32^, ^33^ ^]^ The irradiation by energetic ions, UV light, and plasma, as well as surface capping, was reported to effectively improve the UV emission of ZnO nanostructures, but either require high energy up to MeV or cause rougher surface.^[^ ^20^, ^34^, ^35^, ^36^, ^37^, ^38^, ^39^ ^]^ In addition to the aforementioned roughness issues, the role of oxygen vacancy especially at the outer surface and the lattice distortions at deep sites are underestimated for PL enhancement and left unresolved. Therefore, an economically viable, non‐invasive, and long‐term stable strategy to post‐tackle these imperfections is urgently needed.

Herein, we demonstrate X‐ray irradiation a non‐invasive and effective approach to cure the surface oxygen vacancy of Ga‐doped ZnO microwire, as well as perfectly relax the defective lattice orientation. By recording the photoluminescence signals that reflect the pristine bandgap and defect‐related emissions, we find that moderate X‐ray irradiation (doses ≤150 Gy) can induce a substantial PL augmentation, with a maximized PL value at doses = 150 Gy, which approaches ninefold increment and remains ultra‐stable even after 180‐day air exposure. The PL signal starts to reduce at higher X‐ray doses and saturates at ≥3.3 times the PL value of the un‐irradiated one. In combination with X‐ray photoelectron spectroscopy and varying surrounding atmosphere analysis, oxygen, and water molecules are revealed to dominate the X‐ray‐induced PL enhancement by reconstructing the oxygen vacancy at the surface and shallow subsurface of Ga‐doped ZnO microwire. In addition, the cross‐section transmission electron microscope (TEM) images of irradiated nanowires show that the microwire lattice relaxes close to the ideal value, even for the interior region. Due to the minimized surface oxygen vacancy and lattice relaxation, the as‐irradiated single Ga‐doped ZnO microwire emits highly‐tunable visible lights under a biased voltage, showcasing their great potentials in various micro‐ and nano‐optoelectronic devices, especially for the service scenarios with X‐ray involvements.

## 2 Results and Discussion

**Figure 1:**
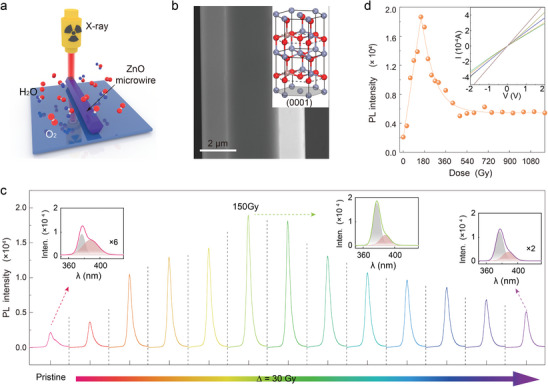
The PL evolution of Ga‐doped ZnO microwire under different X‐ray irradiations. a) Schematic diagram of X‐ray irradiation of Ga‐doped ZnO microwire. b) SEM image of Ga‐doped ZnO microwire and corresponding hexagonal wurtzite crystal model (inset). c) The near‐band edge PL emission of Ga‐doped ZnO microwire (centered at ≈378 nm, also called the ultraviolet peak (UV) peak) varies with irradiation doses. The insets are Gaussian fitting curves of 378 nm peaks at 0, 150, and 420 Gy, from left to right. The X‐axis is wavelength (λ). d) The variation of peak value centered at ≈378 nm under different X‐ray irradiation doses, inserted with the current–voltage plots of as‐growth and irradiated microwires.

**Figure** **1**a sketches the X‐ray irradiation of a single Ga‐doped ZnO microwire under a controllable atmospheric environment. The microwire with a regular hexagonal cross‐section plane and shiny surface (Figure 1b) from chemical vapor deposition was selected (please see Figure S1, Supporting Information for optical and SEM images) and mounted onto a silicon wafer (Si with 300 nm oxide layer) by two indium particles. The irradiation process was conducted by our homemade setup (please see Figure S2, Supporting Information), in which the microwave was placed right below the X‐ray source, and the duration time was adopted to calculate the doses. As shown in the inset crystal model (Figure 1b), the microwire possesses a hexagonal wurtzite structure, with blue‐gray, pink, and red balls representing Zn, Ga, and O atoms, respectively. Initially, the photoluminescence (PL) intensity (centers at ≈378 nm) of as‐grown Ga‐doped ZnO microwire was estimated to be ≈2000. The UV peak could be well fitted by two individual peaks (center at ≈377 and ≈388 nm) with an intensity ratio of 0.90. The former originates from the bandgap (≈3.29 eV) recombination while the latter peak (∼3.19 eV) could be ascribed to the lattice defects in the microwire.^[^ ^40^ ^]^ From the Gauss‐fitting of visible PL spectra, three emission peaks arise centered at (2.03 ± 0.03) eV, (2.16 ± 0.01) eV, and (2.38 ± 0.02) eV, corresponding to the recombination of an electron in zinc interstitial with a hole in singly ionized oxygen vacancy,^[^ ^20^, ^41^ ^]^ the transition energy between the conduction band and the *V*o (+2/0) level,^[^ ^20^, ^41^ ^]^ and the zinc vacancy(*V*_Zn_),^[^ ^42^ ^]^ respectively (more details, see Figure S6, Supporting Information). After different X‐ray irradiations with dose interval of 30 Gy, the PL intensity of Ga‐doped ZnO microwire gradually increases and reaches the maximum point (≈18 000) at the X‐ray doses of ≈120–150 Gy, about nine times more than the pristine PL intensity, which is verified to be stable even after 180 days (see Figure S3, Supporting Information). Afterward, the PL intensity starts to decline with growing X‐ray irradiation doses and tends to be stabilized (≈6700) after a dose of ≈540 Gy. The intensity ratio between bandgap‐related (center at ≈377 nm) and defect‐related (center at ≈388 nm) peaks by Gaussian functions, is calculated to range from ≈0.90 at 0 Gy, ≈3.12 at 150 Gy to ≈2.12 at 420 Gy (see Figure S4 and Table S1, Supporting Information for more details). This variation corresponding to the defect proportion also occurs within extra microwires (see Figure S5, Supporting Information for more details) and the origins of the saturated PL spectrum will be discussed in a later section. In addition, the bandgap‐related PL peak (centers at ≈377 nm, Figure 1d) exhibits a similar evolution trend with the overall PL peak shown in Figure 1c. The stabilized bandgap‐related PL intensity at higher X‐ray doses also suggests that X‐ray irradiation is a mild protocol and introduces no extra defects within ZnO microwires. As a proof‐of‐concept demonstration, we also collected the current‐voltage curve of Ga‐doped ZnO microwire before and after 30 Gy X‐ray irradiation (inset in Figure 1d) and the electrical resistance of irradiated one increase to ≈4.6 kΩ from ≈3.7 kΩ, suggesting the reduction of lattice defects and other feasible gap states by suitable X‐ray irradiation (see Figure S7, Supporting Information for more resistance data).

**Figure 2:**
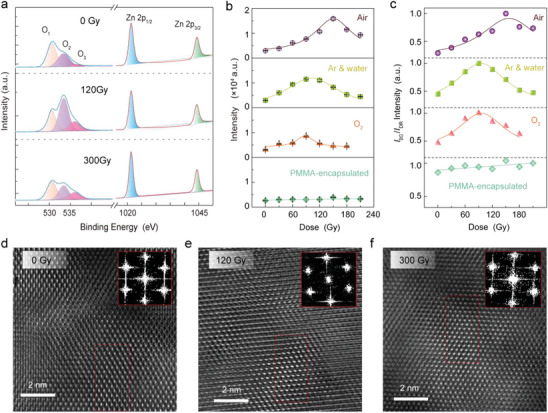
Characteristics of X‐ray irradiated Ga‐doped ZnO microwire. a) XPS spectra of *O* 1s and *Zn* 2p of Ga‐doped ZnO microwires irradiated at 0, 120, and 300 Gy, respectively. b) Plots of X‐ray doses and PL intensity (centers at ≈378 nm) under four‐atmosphere irradiations. PMMA here represents Polymethyl Methacrylate. c) The evolution of *I*_BG_/*I*_DR_ ratio as a function of X‐ray doses under four‐atmosphere irradiations. For clarification, the PL peak at 378 nm is denoted as the bandgap peak (BG) and the at 530 nm is defined as the defect‐related peak (DR). d–f) TEM images of the samples irradiated at 0, 120, and 300 Gy doses, respectively. The inset is the corresponding Fast Fourier Transform (FFT) pattern.

To probe the origin of remarkable PL enhancement, we adopted X‐ray photoelectron spectroscopy (XPS) to monitor the elemental variation and binding energy changes of Ga‐doped ZnO microwire as a function of varying irradiation doses (**Figure** **2**a, from top to bottom panels, 0, 120, and 300 Gy, see Table S2, Supporting Information for the elemental analysis). It is seen that the O 1s peaks in three samples are asymmetric and could decompose into three peaks (*O*_1_ at 530.3 eV, *O*_2_ at 531.7 eV, and *O*_3_ at 533 eV) corresponding to O^2−^ ions, the oxygen of surface hydroxyl (OH) and the adsorbed oxygen at the microwire surface, respectively.^[^ ^43^, ^44^, ^45^, ^46^, ^47^ ^]^ These results evidence the defective nature of Ga‐doped ZnO microwire grown from the CVD method, in good agreement with previous reports.^[^ ^42^ ^]^ In the aspect of Zn 2p spectrum that split into *Zn* 2p_1/2_ (≈1044.33 eV) and 2p_3/2_ (≈1021.3 eV) for pristine Ga‐doped ZnO microwire,^[^ ^48^ ^]^ these two binding energies are seldom shifted after 120 Gy and 300 Gy irradiations, indicating the X‐ray robustness of zinc element. To be quantitative, the stoichiometric ratio of zinc and oxygen (especially the *O*_1_) element was calculated to evaluate the X‐ray effectiveness of crystal lattice perfection (see Table S2, Supporting Information for more details). The *O*_1_/Zn stoichiometric ratio of pristine Ga‐doped ZnO was determined to be ≈82.97%, revealing the massive existence of oxygen vacancy (≈17.03%). The ratio respectively ascends to ≈91.26% and ≈84.04% after 120 and 300 Gy X‐ray irradiations, in line with the PL evolution trend. This consistency proves the X‐ray‐assisted elimination of oxygen vacancy at the microwire surface and topmost few layers, considering the oxygen diffusion limitation and penetration depth of the X‐ray source for XPS characteristics.^[^ ^49^ ^]^ We also conducted a series of XPS experiments at different irradiation doses in air environments (Figure S8a and Table S3, Supporting Information), which is consistent with the aforementioned discussion. We further conducted the electron paramagnetic resonance (EPR) measurement to probe the density of oxygen vacancies (see Figure S9, Supporting Information for more details). The reduced EPR signal at g = 2.003 after 150 Gy X‐ray irradiation reconfirms the X‐ray‐assisted elimination of oxygen vacancies,^[^ ^50^, ^51^ ^]^ coincided with the XPS characteristic of pristine and irradiated Ga‐doped ZnO microwires. Theoretically, surface oxygen vacancy is highly correlated with the band structure and PL emissions, and mild X‐ray irradiation energetically favors the elimination of surface oxygen vacancy (see Figures S10–S12, Supporting Information).

We next focused on the feasible mechanism of irradiation‐enhanced PL spectra by precisely controlling the surrounding atmospheres during X‐ray exposure, including atmosphere (≈25 °C and ≈50% relative humidity), vapor‐carried argon (15% H_2_O_(g)_/Ar, ≈25 °C), oxygen environment (oxygen content is ≈93%, ≈25 °C), and a gas‐free environment encapsulated by a 200‐nm‐thick PMMA layer. For intuitive comparison, four Ga‐doped ZnO microwires with PL intensities of ≈3000 were selected here (Figure 2b) and one microwire exhibits similarly first increases and then decreases PL tendency (maximized intensity ≈15 000 at 150 Gy) as functions of elevated irradiation doses in air atmosphere. For the samples after vapor‐carried argon (15%H_2_O_(g)_/Ar) or oxygen (oxygen content is ≈93%) irradiations, both PL intensities approached the peak value at 90 Gy but with fewer values of ≈11 000 and ≈8600, respectively, with respect to the PL data under air irradiation. This minor deviation may come from a lower concentration of single molecules and reversely verified the synergistic effect of oxygen and water molecules in PL enhancement. For the sample with a PMMA encapsulation layer, without any available gas molecules during X‐ray irradiation, the PL intensity barely changed, and highlights the necessity of oxygen and water molecules toward X‐ray‐assisted PL enhancement. XPS data of different irradiation doses under oxygen and vapor‐carried argon are presented in Figures S8b,c and Tables S4 and S5 (Supporting Information). Normalized by the bandgap (BG) PL peak at 378 nm, the *I*_BG_/*I*_DR_ ratio (Figure 2c) evolves almost as identical as the PL peak at 378 nm varies, reconfirming the X‐ray‐assisted surface lattice perfection, i.e., oxygen vacancy reduction.

To monitor the structural variation after different X‐ray irradiation doses, the samples were imaged by a high‐resolution transmission electron microscope (TEM). Figure 2d–f shows the TEM images of microwire samples irradiated at 0, 120, and 300 Gy doses, respectively, with inserted Fast Fourier Transform (FFT) patterns. It is acknowledged that the distribution points of hexagons in the FFT diagram consist of the lattice information along the plane (0001). The red dotted box indicates the enlarged area displayed in Figure S10 (Supporting Information). The lattice constants at three doses of 0, 120, and 300 Gy were determined to be ≈0.274 ± 0.006 nm, ≈0.279 ± 0.003 nm and ≈0.278 ± 0.005 nm, respectively (see Figure S13 and Table S6, Supporting Information). To offer a quantitative image, 24 sets of lattice constants collected from randomly selected spots from the surface of the sample (0 nm) were plotted in histograms (Figure 2h). The full width at half maximum (FWHM) of three fitting curves that reflect the discrepancy of lattice constant were calculated to be 0.0084, 0.0065, and 0.0082 nm (at dose ≈0, 120, and 300 Gy), respectively. The narrower FWHM of 120 Gy irradiated ZnO microwires proves the effective lattice perfection of mild X‐ray irradiations. As a supplement, the reduced charge radiation relaxation rates after mild X‐ray irradiations reconfirm the minimized surface oxygen vacancy and lattice perfections in Ga‐doped ZnO microwires (see Figure S15, Supporting Information).

**Figure 3:**
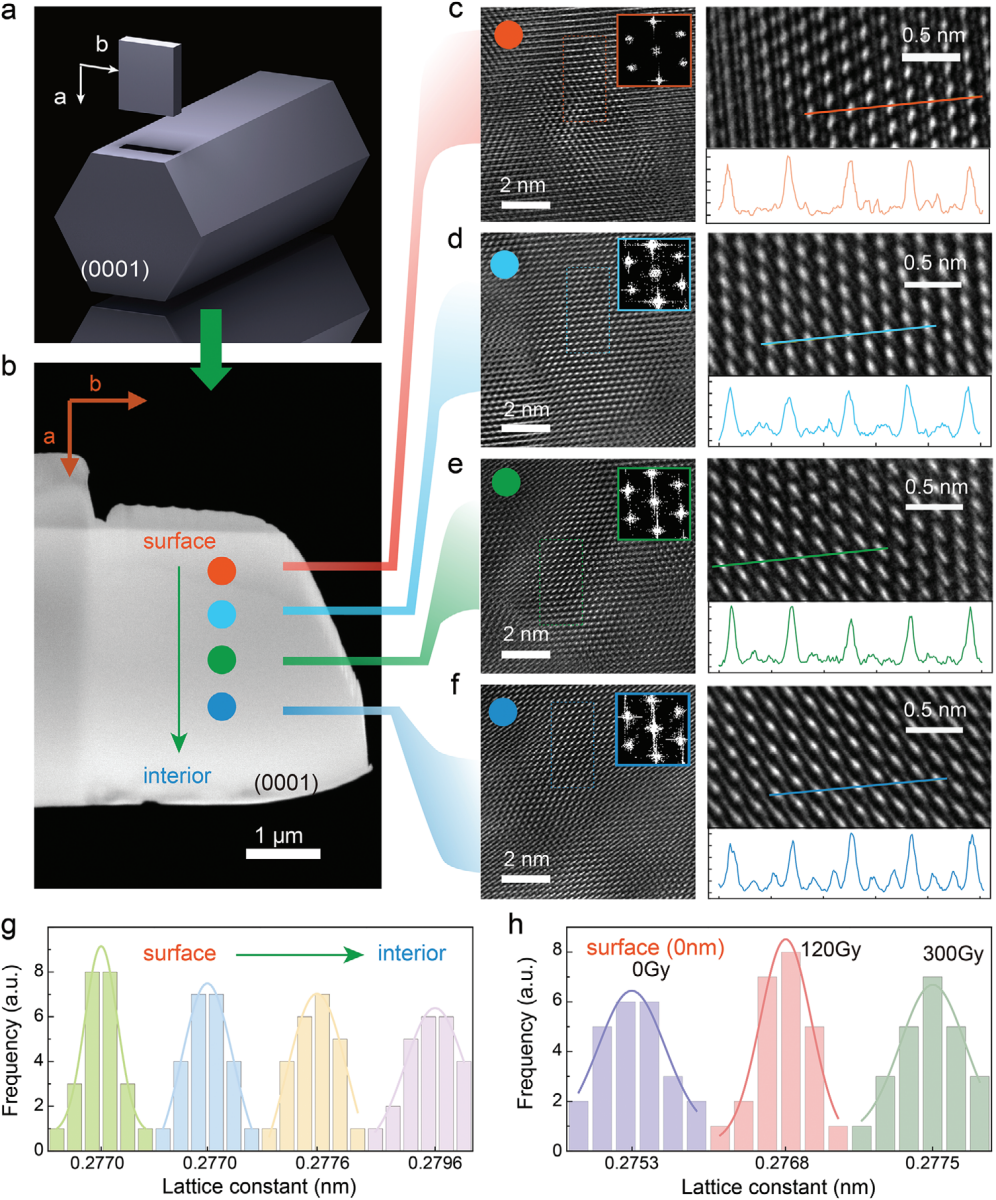
High‐resolution TEM images of Ga‐doped ZnO microwire at different depths. a) Protocol illustration of the FIB slicing. b) SEM image of the sample cross‐section after FIB slicing. c–f) Large‐scale and zoom‐in TEM images of Ga‐doped ZnO microwire at four representative regions. The corresponding line profiles show the spatial lattice structure along one high‐symmetry orientation. g) Histograms of lattice constant distributions of Ga‐doped ZnO microwire at four regions under a dose of 120 Gy. h) The lattice constant distributions of Ga‐doped ZnO microwire surface (a = 0 nm, Figure 3b) at different X‐ray irradiation doses.

X‐ray is a high‐frequency energy wave without heavy ions that may penetrate through the surface of Ga‐doped ZnO microwire into the interior region to modify the lattice structure and even the crystallinity and may also improve the PL emission. To verify the lattice structure at different depths, a focused ion beam (FIB) was employed to slice the sample along the (0001) plane (**Figure** **3**a) and obtained a rectangular slice (Figure 3b) with arrows pointing from the surface to the interior regions of the sample. Figure 3c–f shows the corresponding atomic‐resolution TEM images collected from four regions (sample surface, 0.5, 2.0, 4.0 µm away from the surface) at a dose of 120 Gy. Well‐ordered atoms are clearly observed and the inserted FFT images generate lattice constants of 0.277 ± 0.004 nm, 0.276 ± 0.001 nm, 0.274 ± 0.001 nm and 0.276 ± 0.010 nm along three high‐symmetry orientations (see Table S7, Supporting Information). The four‐line profiles show the ordered atoms but with obvious deviations in brightness and spatial locations, implying the persistence of lattice distortions even after 120 Gy irradiation. To offer a quantitative image, 24 lattice constants collected from 24 randomly selected spots for each specified region were plotted in histograms (Figure 3g). The FWHMs of four fitting curves that reflect the discrepancy of lattice constant were calculated to be ≈0.0055, 0.0087, 0.010, and 0.010 nm, respectively. It is indicated that the lattice relaxation becomes remarkably obvious at the surface and gradually reduces for the inner region of Ga‐doped ZnO microwire. In comparison with the lattice constant of the pristine sample (Figure 3h), low doses of X‐ray irradiations (<120 Gy) properly relax the lattice and diminish the intrinsic lattice distortion. In addition, the irradiation‐irrelevant angle‐resolved Raman and X‐ray diffraction features re‐confirm the non‐invasive nature of X‐ray irradiation toward PL enhancement (Figures S16 and S17, Supporting Information).

**Figure 4:**
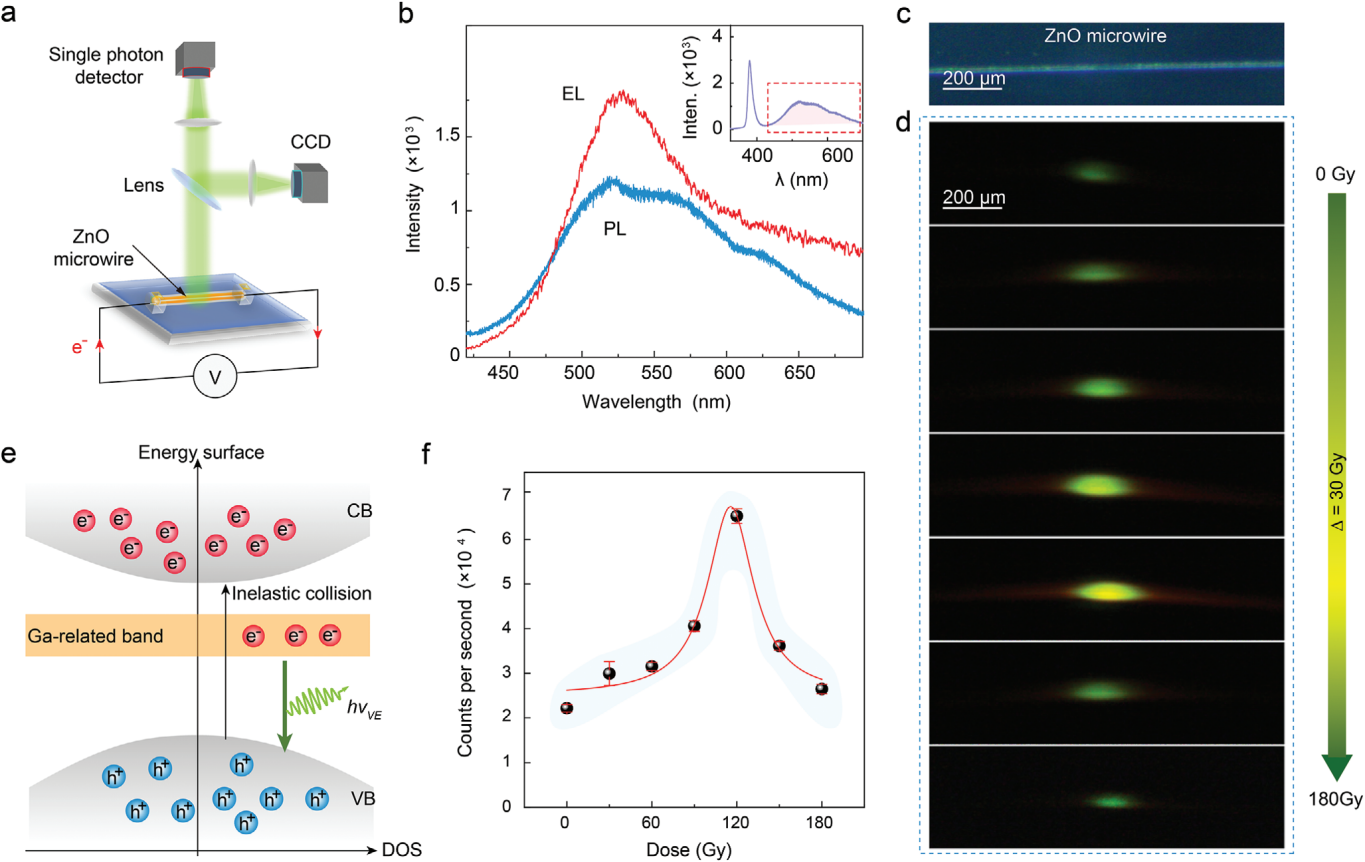
Electroluminescence of Ga‐doped ZnO microwire after different X‐ray irradiation doses. a) Experimental setup for recording electroluminescence and single photon emission rate. b) The EL of Ga‐doped ZnO microwire, with respect to the corresponding PL spectra. The inserted curve is the full‐range PL spectrum. (c) Optical image of selected Ga‐doped ZnO microwire. d) EL images at different irradiation doses with the irradiation dose interval is 30 Gy, with the same voltage and injection current (18 V and 6.1 mA). e) Schematic diagram of the proposed electroluminescence process. After absorbing efficient energy, electrons are excited into the conduction band and then the non‐equilibrium electrons level relaxes and transit to the Ga‐related bands. Finally, radiative recombination occurs between the electrons in the Ga‐related bands and the holes in the valence bond, emitting bright visible light at the center of the microwire. f) The plots between the number of emitted photons per second and the irradiation doses. The red line is the Lorentz fitting.

The reduced surface oxygen vacancy and lattice relaxation almost throughout the microwire promote Ga‐doped ZnO microwire's great potential for applications as electroluminescent (EL) light sources. **Figure** **4**a depicts the EL measurement of a biased Ga‐doped ZnO microwire, with a single‐photon counter capturing the emitted photons and a CCD camera, respectively. The EL spectrum (red curve, Figure 4b) of a fresh Ga‐doped ZnO microwire (Figure 4c) exhibits a prominent peak at ≈520 nm, similar to the defect‐related PL signal (blue curve, Figure 4b). The voltage was set at 18 V (injection current ≈6.1 mA), and the brightness and spot size of the emitted light increased monotonically for X‐ray irradiation doses below 120 Gy. The enhancement of defect‐related EL lights was ascribed to the competition of oxygen vacancies and Ga dopants. The Ga dopant with concentration <1% forms a Ga‐related band with a ZnO bandgap. With increasing X‐ray irradiation, the nonuniform oxygen vacancy at the surface that may swell the band edge^[^ ^52^ ^]^ or favor heat generations is gradually reduced while the Ga‐related band because of Ga dopants dominate the electroluminescence process. The bias‐excited electrons in the conduction band easily relax into the Ga‐related band. After that, electrons from the Ga‐related band recombine with the holes in the valence band, meanwhile, emitting green lights. The reduction of oxygen vacancy enables well‐defined band edges, which favor more carriers involved in the electroluminescence process and consequently enhance the emission of green lights. As a supplement, the variation of photon emitting rate with irradiation doses was recorded in Figure 4f. The emitting rate is increased to 66 000 per second after 120 Gy X‐ray irradiation from an initial 20 000 per second (pristine sample without irradiation), demonstrating X‐ray is one promising approach for boosting ZnO microwires in practical optoelectrical devices.

## 3 Conclusion

In summary, we demonstrate a giant photoluminescence enhancement of Ga‐doped ZnO microwire by X‐ray irradiation in ambient conditions. The irradiation of CVD‐grown Ga‐doped ZnO microwire under moderate doses (≤150 Gy) favors the elimination of surface oxygen vacancy and lattice relaxations throughout the microwires, evidenced by X‐ray photoelectron spectrum and cross‐section transmission electron microscopic characteristics. These variations significantly contribute to the band edge electron‐hole recombination and ninefold magnify the following photoluminescence. The enhanced photoluminescence barely decays during air exposure for 180 days. As a proof‐of‐concept demonstration, we further show amplified electroluminescence of Ga‐doped ZnO microwire by more than three times after mild X‐ray irradiation. The X‐ray irradiations under a controlled atmosphere suggest the X‐ray‐assisted elimination of oxygen vacancy at the microwire surface and topmost few layers while the barely unchanged visible PL emission under increased X‐ray dose reveals no extra defects induced by X‐ray irradiation. The X‐ray irradiation method is compatible with present semiconductor processing technology and may enable the implantation of various ZnO nanostructures into various optoelectronic devices, such as UV LEDs, photodetectors, and solar cells.

## 4 Experimental Section

### Growth of Ga‐Doped ZnO Microwires

The hexagonal cylindrical Ga‐doped ZnO microwires were synthesized by a homemade chemical vapor deposition, akin to the previous report.^[^ ^42^, ^53^ ^]^ To be more specified, the mixed precursors of ZnO, Ga_2_O_3_, and graphite powders with high purity in the weight proportion of 9:1:10 were placed in a corundum boat (length × width × depth = 20 cm × 2 cm × 1.5 cm). A 2 cm × 2 cm cleaned silicon wafer was placed on top of the power mixture to collect the ZnO products. Both the boat and wafer were together loaded into quartz tube, away from the center heating zone. A constant flow of argon (Ar) (99.99%, 120 sccm) was introduced into the tube furnace as the protecting gas. After the furnace was programmed to heat up to 1100 °C, the precursor and silicon wafer were moved into the central heating zone and held for 60 min in the presence of 10% oxygen (O_2_). After natural cooling to room temperature, one may collect numerous Ga‐doped ZnO microwires around and on top of a silicon wafer, where Ga_2_O_3_ is used as a precursor and Ga atoms are in situ doped into ZnO microwire, with the doping concentration <1% (more details, see Figures S14 and S15, Supporting Information). For this concentration, the lattice distortion caused by the Ga dopant can be ignored.^[^ ^20^, ^42^ ^]^

### Structural and Spectral Characterizations

The X‐ray emitter (XRB‐160 industrial) was employed as the X‐ray source with a tube pressure of 160 kV, tube flow of 2 mA, the W target, and dose rate of 0.71 Gy min^−1^. The irradiation dose rate was pre‐calibrated by a Fricke dosimeter and consequently determined to be ≈3.37 Gy min^−1^ for ZnO microwires (Figure S2, Supporting Information). The prepared Ga‐doped ZnO microwires were carefully sliced and characterized by double‐beam scanning electron microscopy (SEM, LYRA3 GMU, TESCAN Orsay Holding). For transmission electron microscopy (TEM) samples, the Ga‐doped ZnO microwires were first machined by focused ion beams (FIB, Scios 2 HiVac QUANTAX 200 with XFlash6|60, Thermo Fisher Scientific Bruker Corporation) and then analyzed by double spherical aberration‐corrected transmission electron microscopy (STEM, Spectra 300, Thermo Fisher Scientific). The surface composition and chemical states were qualitatively and quantitatively analyzed by X‐ray photoelectron spectroscopy (ESCALAB Xi+, Thermo Fisher Scientific, with ≈10 nm detection depth). The electron paramagnetic energy spectrum (EPR, Bruker A300) was also adopted to evaluate the oxygen vacancies. Room‐temperature PL and Raman measurements were performed using a confocal laser Raman spectrometer (LabRAM HR Evolution) with 325 and 532 nm excitation lasers, respectively. The former was adopted for the PL excitation source while the latter was selected for the Raman spectrum. Time‐resolved photoluminescence (TRPL) measurements were performed using a steady‐state transient fluorescence spectrometer (FLS1000 Edinburgh Instruments at 320 nm). The counting rate of electroluminescence was captured by a single photon counting system (PicoHarp 300).

## Conflict of Interest

The authors declare no conflict of interest.

## Supporting information



Supporting Information

## Data Availability

The data that support the findings of this study are available from the corresponding author upon reasonable request.
