# Supplementary material for: Giant Photoluminescence Enhancement of Ga‐Doped ZnO Microwires by X‐Ray Irradiation
Source: Adv Sci (Weinh). 2024 Nov 25;12(3):2407144. doi: 10.1002/advs.202407144 (PMC11744572; doi:10.1002/advs.202407144)
Supplement: Supplementary file 1 — Supporting Information [file ADVS-12-2407144-s001.docx]

**Supplementary Information**

**Giant Photoluminescence Enhancement of Ga-doped ZnO Microwires by X-ray Irradiation**

Siyuan He^1,2^, Shuiyan Cao^1,3,4*^, Ying Liu^1,2^, Wenfa Chen^1,2^, Pin Lyu^1,2^, Weidian Li^1^, Jincheng Bao^1^, Wenhui Sun^1^, Caixia Kan^1,3,4^, Mingming Jiang^1,3,4^, Yanpeng Liu^1,2,4*^

**Table of Contents**

S1. Optical and SEM images of as-grown Ga-doped ZnO microwires S2

S2. Experimental setup of X-ray irradiation S3

S3. Long-term stability test S4

S4. PL fits of Ga-doped ZnO microwire after varying X-ray irradiations S5

S5. Additional PL enhancements of Ga-doped ZnO samples S7

S6. The de-convoluted PL spectra of Ga-doped ZnO microwire with varied irradiation doses. S9

S7. The *I-V* plots of Ga-doped ZnO microwire under varied irradiation doses. S11

S8. XPS characteristics of different-dose X-ray irradiated ZnO samples S12

S9. XPS characteristics of different-dose X-ray irradiated ZnO samples under atmosphere. S13

S10. EPR spectra of Ga-doped ZnO microwire irradiated at 0 Gy and 150 Gy. S16

S11. Density functional theory (DFT) calculation. S17

S12. TEM images and profiles of Ga-doped ZnO microwires at three irradiation doses. S21

S13. Determining the lattice structure from One representative FFT image S22

S14. Normalized time-resolved photoluminescence (TRPL) spectra. S23

S15. Raman signals of pristine and irradiated Ga-doped ZnO microwires S24

S16. XRD of irradiated Ga-doped ZnO microwires. S25

S17. Description of Ga-doping concentration S26

S18. Comparison of different methods S28

References S30

# S1. Optical and SEM images of as-grown Ga-doped ZnO microwires

**
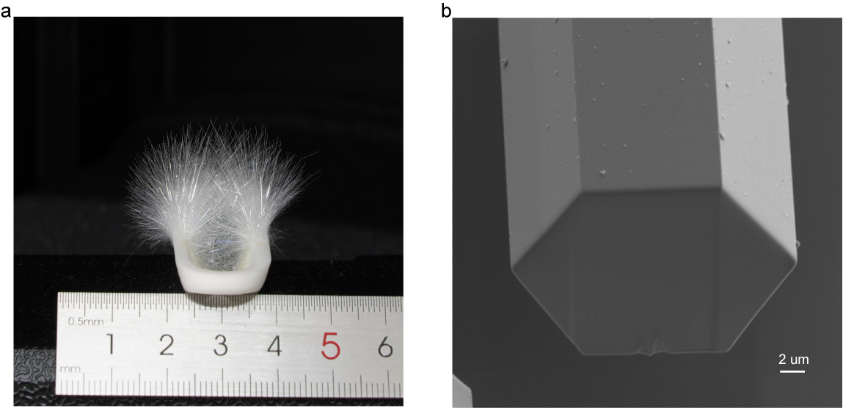
**

**Figure S1. Optical and SEM images of Ga-doped ZnO microwires synthesized by chemical vapor deposition.** (a) An optical image of Ga-doped ZnO microwires right after CVD growth. The sparking microwires atop the crucible boat indicates the high crystalline of as-grown samples. The microwire length lies in the order of millimeter and the length of several microwires surpass ten millimeters. (b) One representative SEM image of Ga-doped ZnO microwires showing well-defined hexagon with smooth surface.

# S2. Experimental setup of X-ray irradiation


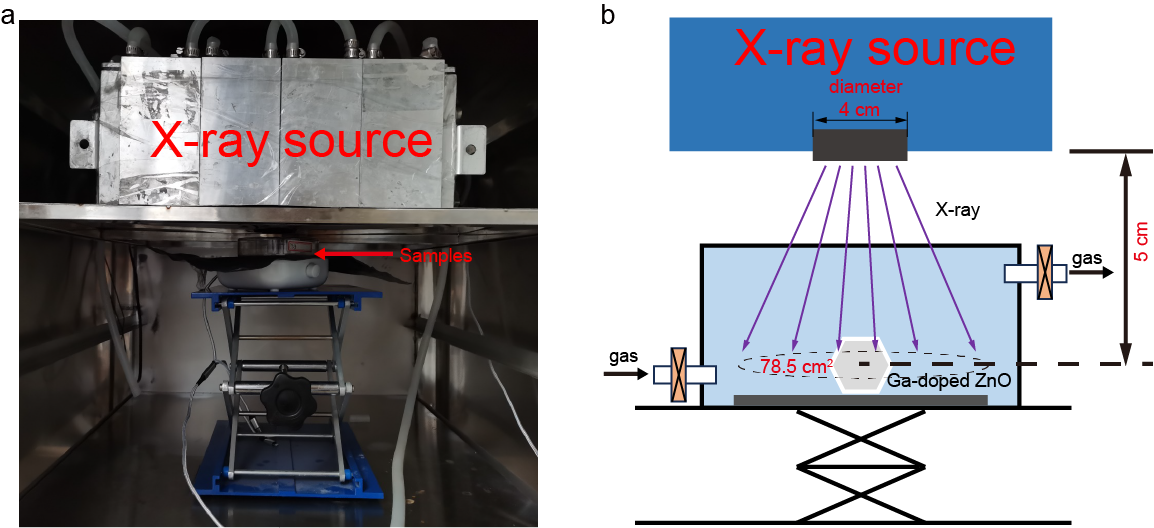


**Figure S2. X-ray irradiation apparatus and schematic diagram of irradiation under controlled atmosphere.** (a) The image of our home-made irradiation equipment. (b) The sketched irradiation process under controlled atmosphere.

The X-rays were emitted from a circular emission port with a diameter of ~4 cm and the distance between the port and ZnO microwire was set to be 5 cm, which produce a circular irradiation area of ~ 78.5 cm^2^. To be quantitative, The X-ray dose rate was calibrated by a Fricke dosimeter based on the absorbed dose of water. Typically, The dose rate of our setup was determined to be ~0.71 Gy/min, which follows the dosimetry standards and can be used for the irradiation experiment. Therefore, the dose rate here could be calculated by the absorbed dose rate of water. In accordance to the electron density ratio (~4.74) between ZnO and H_2_O, the actual irradiation dose of ZnO microwire is determined to be ~3.37 Gy/min. The atmosphere is achieved by a sealed and transparent box with inlet and outlet valves. Oxygen or vapor-carried argon enter the box through a pipe with a valve.

# S3. Long-term stability test


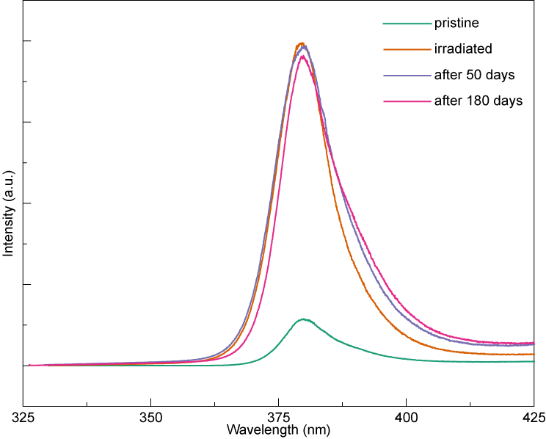


**Figure S3. Ambient stability test of irradiation-enhanced PLs.** The PL enhancements are well-maintained after sample air exposures for 50 days and even 180 days. During the test, the sample was kept in laboratory environment (temperature between 20℃-25℃, relative humidity ~ 45% - 60%).

# S4. PL fits of Ga-doped ZnO microwire after varying X-ray irradiations

**
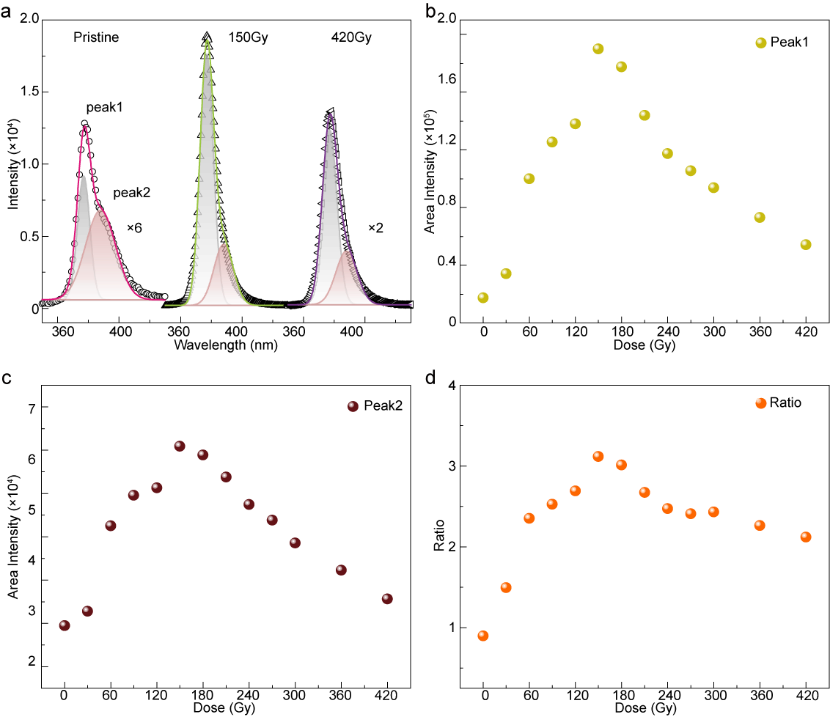
**

**Figure S4. PL fittings of** **Ga-doped ZnO microwires.** (a) Spectral fittings of three PLs at 0 Gy, 150 Gy and 420 Gy irradiation doses, respectively. Each PL peak could be well fitted by two individual peaks (peak 1 centers at ~377 nm and peak 2 centers ∼388 nm) rather than one single peak, revealing the defective nature of CVD-growth Ga-doped ZnO microwires. To be more specified, the former one originates from the bandgap (~3.29 eV) recombination while the latter peak could be ascribed to lattice defects^1^ in the microwire. (b-c) The evolution of peaks 1 and 2 as function of varied irradiation doses. (d) The corresponding peak1/peak2 area ratio at each irradiation dose. Please refer Table 1 for more fitting details.

From 0 Gy to 420 Gy, each PL peak was well fitted into two Gaussian peaks. Peak 1 centers at ~377 nm and peak 2 centers ∼388 nm. According to the Gaussian fitting, the area of each peak and then the area ratio of peak 1 and peak 2 is obtained. The variation trend was consistent with that of PL intensity, reaching its maximum value (~3.12) at dose of ~150 Gy.

**Table S1.** **Detailed fitted peak locations and peak area ratios.**

| Doses | Pos. of peak1 (nm) | Pos. of peak2 (nm) | Area of peak1 (a.u.) | Area of peak2 (a.u.) | Peak1/ peak2 ratio |
| --- | --- | --- | --- | --- | --- |
| 0Gy | 377 | 388 | 17477.50 | 19454.09 | 0.90 |
| 30Gy | 377 | 388 | 34053.40 | 22770.49 | 1.50 |
| 60Gy | 377 | 388 | 99987.28 | 42527.22 | 2.35 |
| 90Gy | 377 | 388 | 125294.81 | 49571.2 | 2.53 |
| 120Gy | 377 | 388 | 138042.41 | 51312.13 | 2.69 |
| 150Gy | 377 | 388 | 189885.38 | 60908.17 | 3.12 |
| 180Gy | 377 | 388 | 177386.79 | 58871.53 | 3.01 |
| 210Gy | 377 | 388 | 143791.78 | 53783.02 | 2.67 |
| 240Gy | 377 | 388 | 117295.58 | 47453.00 | 2.47 |
| 270Gy | 377 | 388 | 105528.39 | 43826.22 | 2.41 |
| 300Gy | 377 | 388 | 93743.02 | 38554.83 | 2.43 |
| 360Gy | 377 | 388 | 73049.63 | 32266.46 | 2.26 |
| 420Gy | 377 | 388 | 54315.66 | 25599.94 | 2.12 |

# S5. Additional PL enhancements of Ga-doped ZnO samples

**
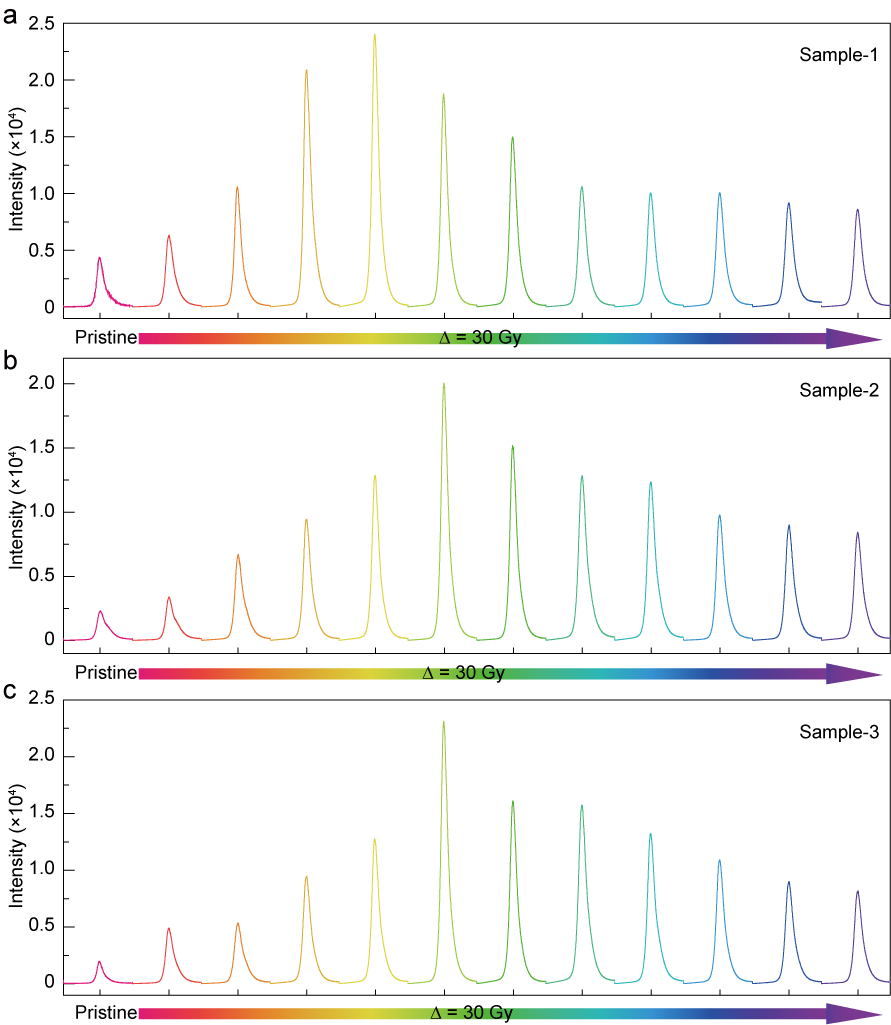
**

**Figure S5. Statistical PL enhancements of three extra ZnO microwires under varied X-ray irradiations.** To show the universality of X-ray irradiation effect, we conducted similar measurements of three additional CVD-growth ZnO samples. After different X-ray irradiations with dose interval of 30 Gy, the PL intensity of sample-1(a) gradually increases and reaches the maximum point (~24000) at the doses of 120 Gy, about six times more than the pristine PL intensity (~4000). Afterward, the PL intensity starts to decline with growing X-ray irradiation doses and tend to be stabilized (~8300) after a dose of ~300 Gy. Similarly, the PL intensity of sample-2 (b) reaches the maximum value (~20000) at the doses of 150 Gy, about nine times more than the pristine PL intensity (~2200). Afterward, the PL intensity starts to decline with growing X-ray irradiation doses and tend to be stabilized (~8300) for the doses ≥ ~360 Gy. For sample-3 (c), the PL intensity maximizes to be ~22000 at the doses of 150 Gy, about ten times more than the pristine PL intensity (2200). After that, the PL intensity starts to decline with growing X-ray irradiation doses and tend to be stabilized (~8100) after a dose of ~360 Gy. It is seen that the PL intensity of all three samples reach the maximum at ~120 – 150 Gy dose and stabilize once the dose surpasses ~360 Gy.

# S6. The de-convoluted PL spectra of Ga-doped ZnO microwire with varied irradiation doses.


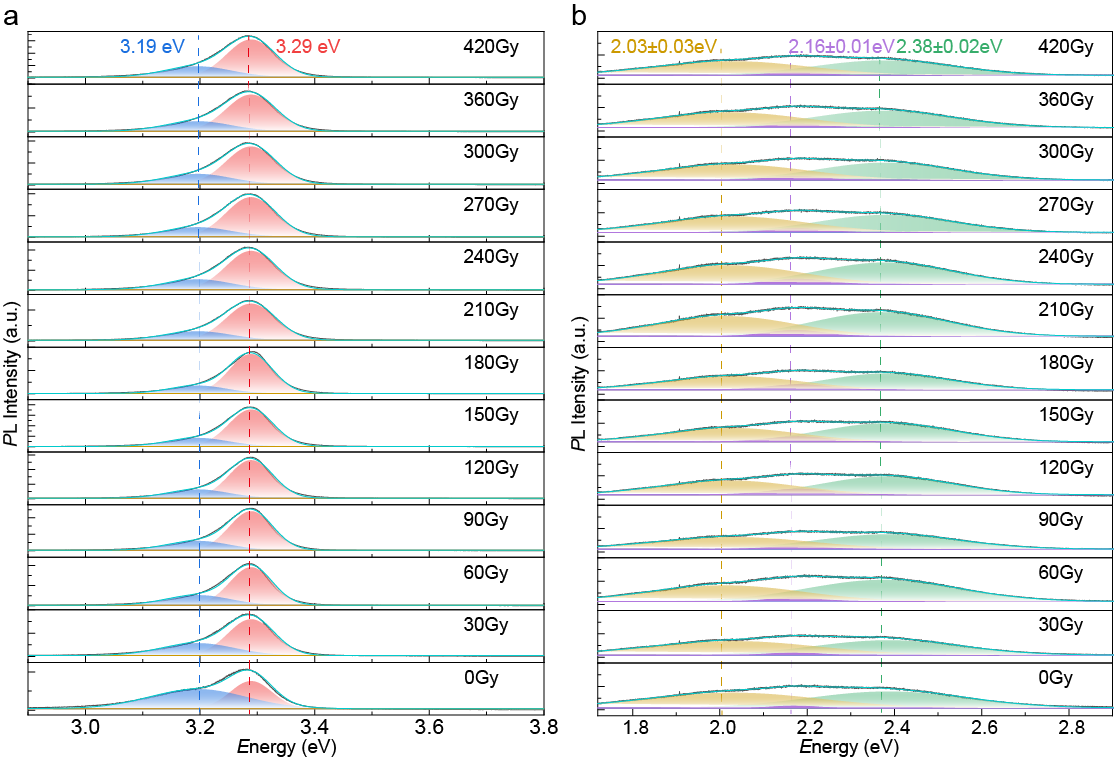

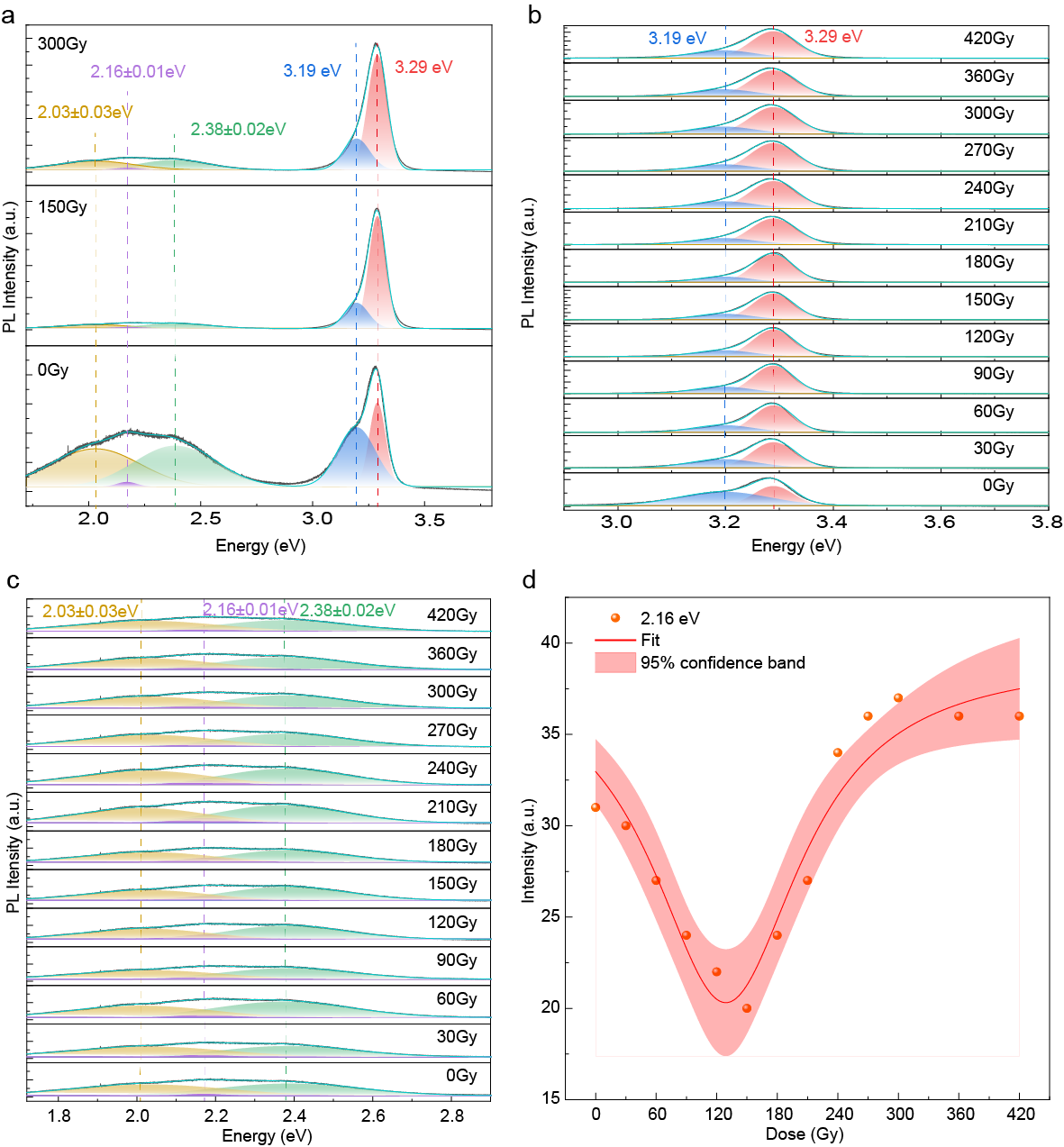


**Figure S6. The de-convoluted PL spectra of Ga-doped ZnO microwire with varied irradiation doses.**(a) The de-convoluted PL spectra at three doses of 0 Gy, 150 Gy and 300 Gy. (b)The de-convoluted UV PL spectra. (c) The de-convoluted visible PL spectra. (d) The area evolution of PL peak at 2.16±0.01 eV varying with irradiation doses.

We performed Gaussian fitting for UV and visible bands in PL spectra, as shown in Figure S6a. The UV peak could be fitted by two individual peaks. One originates from the bandgap (~3.29 eV) recombination, and another peak could be ascribed to the lattice defects^1^ in the microwire (~3.19 eV). From the fitted spectra with Gaussian function, we found that the visible emission bands consist three emission peaks, which centered at (2.03 ± 0.03) eV, (2.16 ± 0.01) eV, and (2.38 ± 0.02) eV, corresponding to the recombination of an electron in Zinc interstitial with a hole in the singly ionized oxygen vacancies^2, 3^, the transition energy between the CB and the *V*_O_ (+2/0) level^2, 3^, and the zinc vacancy(*V*_Zn_)^4^, respectively. The area variation of 2.16±0.01 eV peak with X-ray irradiation dose was further studied, and is shown in the Figure S6d. The PL evolution nicely supports our theoretical mechanism, from which the increased PL emission could be ascribed to the reduced surface oxygen vacancy by mild X-ray irradiation.

# S7. The *I-V* plots of Ga-doped ZnO microwire under varied irradiation doses.


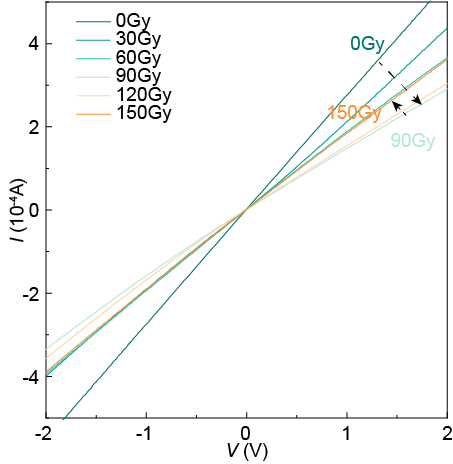


**Figure S7. The current-voltage plots of Ga-doped ZnO microwire with varied irradiation doses.**

With increasing X-ray irradiations, the resistance of microwire exhibits a tendency to increase first and then decrease. The initial resistance of Ga-doped ZnO microwire is ~3.7 kΩ, reaches the maximum value (~6.0 kΩ) after 90 Gy X-ray irradiation and finally reduces to ~5.6 kΩ after 150 Gy X-ray irradiation.

# S8. XPS characteristics of different-dose X-ray irradiated ZnO samples

The XPS spectra of the samples at three doses of 0 Gy, 120 Gy and 300 Gy were analyzed. The O 1s peaks in all three samples are asymmetric and decompose into three peaks (*O*_1_ at 530.3 eV, *O*_2_ at 531.7 eV and *O*_3_ at 533 eV) corresponding to O^2-^ ions, the oxygen of surface hydroxyl (OH) and the adsorbed oxygen at the microwire surface, respectively^5-9^. In the aspect of Zn 2p spectrum, it splits into *Zn* 2p_1/2_ (~1044.33 eV) and 2p_3/2_ (~1021.3 eV) for pristine Ga-doped ZnO microwire^10^. The peak areas and atomic sensitivity factors, the atomic percentages of *Zn*, *O*_1_, *O*_2_, *O*_3_ and relative ratio of *O*_1_ /*Zn* were also calculated and summarized. The relative ratios of lattice oxygen to zinc are less than 1.0, indicating the existed oxygen vacancy at the sample surface.

**Table S2.** **The elemental Zn and O contents determined by XPS.**

| Doses | Percent of Zn (%) | Percent of O_1_ (%) | Percent of O_2_ (%) | Percent of O_3_ (%) | O1/Zn ratio (%) | Percent of oxygen vacancy (%) |
| --- | --- | --- | --- | --- | --- | --- |
| Pristine  (0 Gy) | 37.16 | 30.83 | 21.10 | 10.92 | 82.97 | 17.03 |
| 120 Gy | 26.53 | 24.21 | 37.35 | 11.91 | 91.26 | 8.74 |
| 300Gy | 32.02 | 26.91 | 20.99 | 20.08 | 84.04 | 15.96 |

# S9. XPS characteristics of different-dose X-ray irradiated ZnO samples under atmosphere.

Figure S8 shows the XPS data at multiple radiation doses under different irradiation atmospheres, including air, O_2_ and vapor-carried Ar. To be quantitatively, the elemental Zn and O contents determined by XPS were listed in Table S3-S5. It is seen that the concentration of oxygen vacancy at the sample surface decreases with increased irradiation doses. The surface oxygen vacancies of Ga-doped ZnO microwires can be repaired by small doses of X-ray irradiation. However, under high dose irradiation, oxygen vacancies may be reproduced at the sample surface. By comparing the XPS spectra under different atmospheres, one can conclude that both oxygen and water molecules are of great importance for X-ray-assisted elimination of oxygen vacancy.


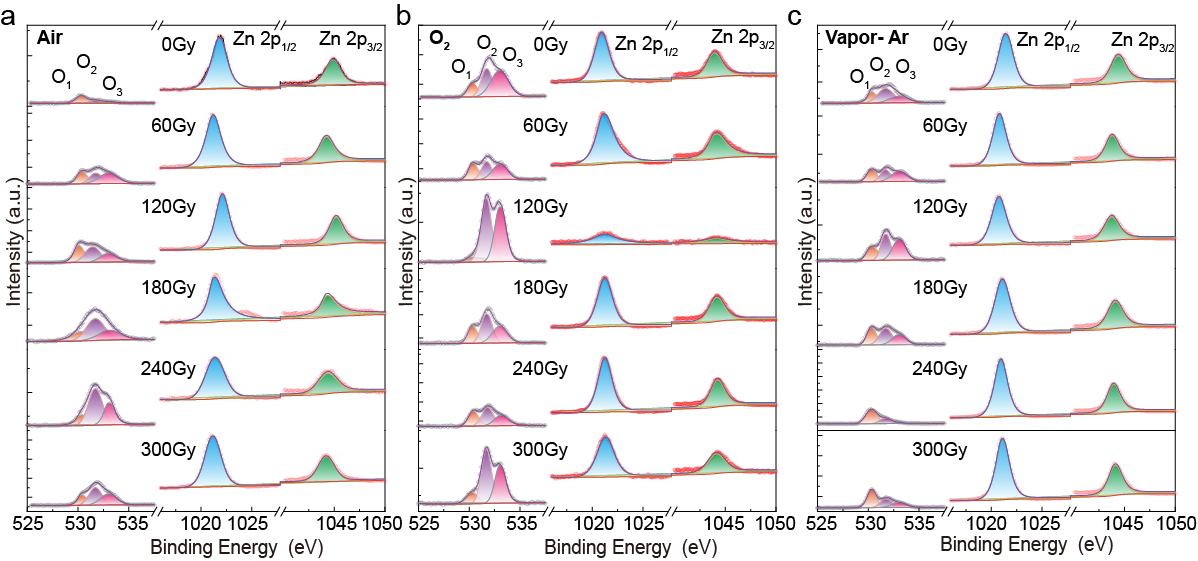


**Figure S8. XPS spectra of Ga-doped ZnO microwire under different atmosphere irradiation.** (a) XPS spectra of *O* 1s and *Zn* 2p of Ga-doped ZnO microwires irradiated under air atmosphere with varied irradiation doses. (b) XPS spectra of *O* 1s and *Zn* 2p of Ga-doped ZnO microwires irradiated under O_2_ atmosphere varies with irradiation doses. (c) XPS spectra of *O* 1s and *Zn* 2p of Ga-doped ZnO microwires irradiated under vapor-carried argon atmosphere varies with irradiation doses.

**Table S3. The elemental *Zn* and *O* contents determined by XPS under Air irradiation.**

| Doses | Percent of Zn (%) | Percent of O_1_ (%) | Percent of O_2_ (%) | Percent of O_3_ (%) | O_1_/Zn ratio (%) | Percent of oxygen vacancy (%) |
| --- | --- | --- | --- | --- | --- | --- |
| Pristine  (0 Gy) | 51.26 | 33.90 | 9.37 | 5.48 | 66.13 | 33.87 |
| 60 Gy | 30.13 | 21.17 | 19.18 | 29.52 | 70.26 | 29.74 |
| 120Gy | 24.59 | 22.84 | 27.84 | 24.73 | 92.88 | 7.12 |
| 180Gy | 21.87 | 20.84 | 36.66 | 20.63 | 95.29 | 4.71 |
| 240Gy | 17.38 | 14.65 | 44.02 | 23.95 | 84.29 | 15.71 |
| 300Gy | 25.93 | 18.48 | 30.88 | 24.71 | 71.27 | 28.73 |

**Table S4. The elemental *Zn* and *O* contents determined by XPS under O_2_ irradiation.**

| Doses | Percent of Zn (%) | Percent of O_1_ (%) | Percent of O_2_ (%) | Percent of O_3_ (%) | O_1_/Zn ratio (%) | Percent of oxygen vacancy (%) |
| --- | --- | --- | --- | --- | --- | --- |
| Pristine  (0 Gy) | 18.17 | 12.90 | 27.96 | 40.96 | 71.00 | 29.00 |
| 60 Gy | 28.83 | 22.34 | 21.91 | 26.93 | 77.49 | 22.51 |
| 120Gy | 6.29 | 5.70 | 45.16 | 42.85 | 90.62 | 9.38 |
| 180Gy | 21.61 | 18.13 | 33.78 | 26.48 | 83.90 | 16.10 |
| 240Gy | 29.07 | 21.44 | 28.97 | 20.52 | 73.75 | 26.25 |
| 300Gy | 14.18 | 10.71 | 42.17 | 32.93 | 75.53 | 24.47 |

**Table S5. The elemental *Zn* and *O* contents determined by XPS under Vapor-Ar irradiation.**

| Doses | Percent of Zn (%) | Percent of O_1_ (%) | Percent of O_2_ (%) | Percent of O_3_ (%) | O_1_/Zn ratio (%) | Percent of oxygen vacancy (%) |
| --- | --- | --- | --- | --- | --- | --- |
| Pristine  (0 Gy) | 31.84 | 17.32 | 33.51 | 17.33 | 54.40 | 45.60 |
| 60 Gy | 32.00 | 20.78 | 20.13 | 27.09 | 64.94 | 35.06 |
| 120Gy | 22.27 | 19.32 | 28.00 | 30.40 | 86.75 | 13.25 |
| 180Gy | 29.48 | 24.83 | 27.46 | 18.23 | 84.23 | 15.77 |
| 240Gy | 45.41 | 37.08 | 15.77 | 1.74 | 81.66 | 18.34 |
| 300Gy | 39.68 | 30.81 | 17.56 | 12.15 | 77.65 | 22.35 |

# S10. EPR spectra of Ga-doped ZnO microwire irradiated at 0 Gy and 150 Gy.


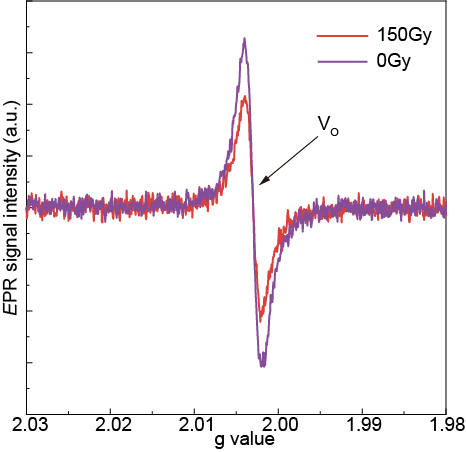


**Figure S9. EPR spectra of Ga-doped ZnO microwire irradiated at 0 Gy and 150 Gy.**

The EPR data of Ga-doped ZnO microwires before (0 Gy) and after irradiation (150 Gy) are shown in the following Figure S9. The unique EPR signal at g=2.003 is related to oxygen vacancies^11, 12^. It indicated that compared with the initial sample, the concentration of oxygen vacancy decreased after 150 Gy irradiation, coincided with the XPS characteristic of pristine and irradiated Ga-doped ZnO microwires.

# S11. Density functional theory (DFT) calculation.

To get more insight into the mechanistic explanations of the enhancement of PL by X-ray irradiation, density functional theory (DFT) calculation was employed to calculate the relevant model. All DFT calculations were carried out using the QUANTUM ESPRESSO plane-wave code ^13, 14^. The Perdew-Burke-Ernzerhof (PBE) exchange-correlation functional within the generalized gradient approximation (GGA) was employed ^15, 16^. Pseudopotentials were sourced from the SSSP library, which is optimized for computational efficiency ^17^. A kinetic energy cutoff of 50 Ry was used for wavefunctions, while 400 Ry was set for charge density. The electronic structure calculations and structural relaxations were converged with thresholds of 7.6 × 10⁻⁹ Ry and 10⁻⁴ Ry/Bohr for the total energy and atomic forces, respectively. To account for strong on-site Coulomb interactions, the DFT + U approach was applied, with Hubbard U parameters fixed at 8 eV for both U_Zn−3d_ and U_O−2p_ states ^18^. The influence of oxygen vacancies on the electronic structure was explored using a ZnO ($10\bar{1}0$) supercell surface consisting of 40 atoms, with a Monkhorst–Pack k-point grid of 7 × 9 × 2 for Brillouin zone sampling. A vacuum spacing of 20 Å is introduced to avoid artificial interactions between the periodic images along the z-direction.

To get more insights, density functional theory (DFT) calculation was employed based on Zn_20_O_20_, Zn_20_O_18_ and Zn_20_O_16_ models, as shown in Figure S10 and Figure S11. For oxygen-vacancy-free Zn_20_O_20_ model, the band structure (Fig. S10d) exhibits a band gap of ~3.29 eV, in good agreement with reported values in previous literatures ^19, 20^. After introducing oxygen vacancy, a few more energy levels appear in the band gap regions (Fig. S10e and 10f) and may involve in the charge excitation and recombination process. As such, the PL performance could be manifested by oxygen vacancies. As can be seen from the total density of states (Fig. S11), the presence of oxygen vacancies swells two band edges and even reduces the electronic bandgap, thereby reducing the intrinsic bandgap photoluminescence. The as-grown Ga-doped ZnO microwires are recognized to contain plentiful oxygen vacancies at their surfaces that affect the PL performances. In this study, the X-ray irradiation was demonstrated to eliminate these oxygen vacancies and lead to the PL enhancements.


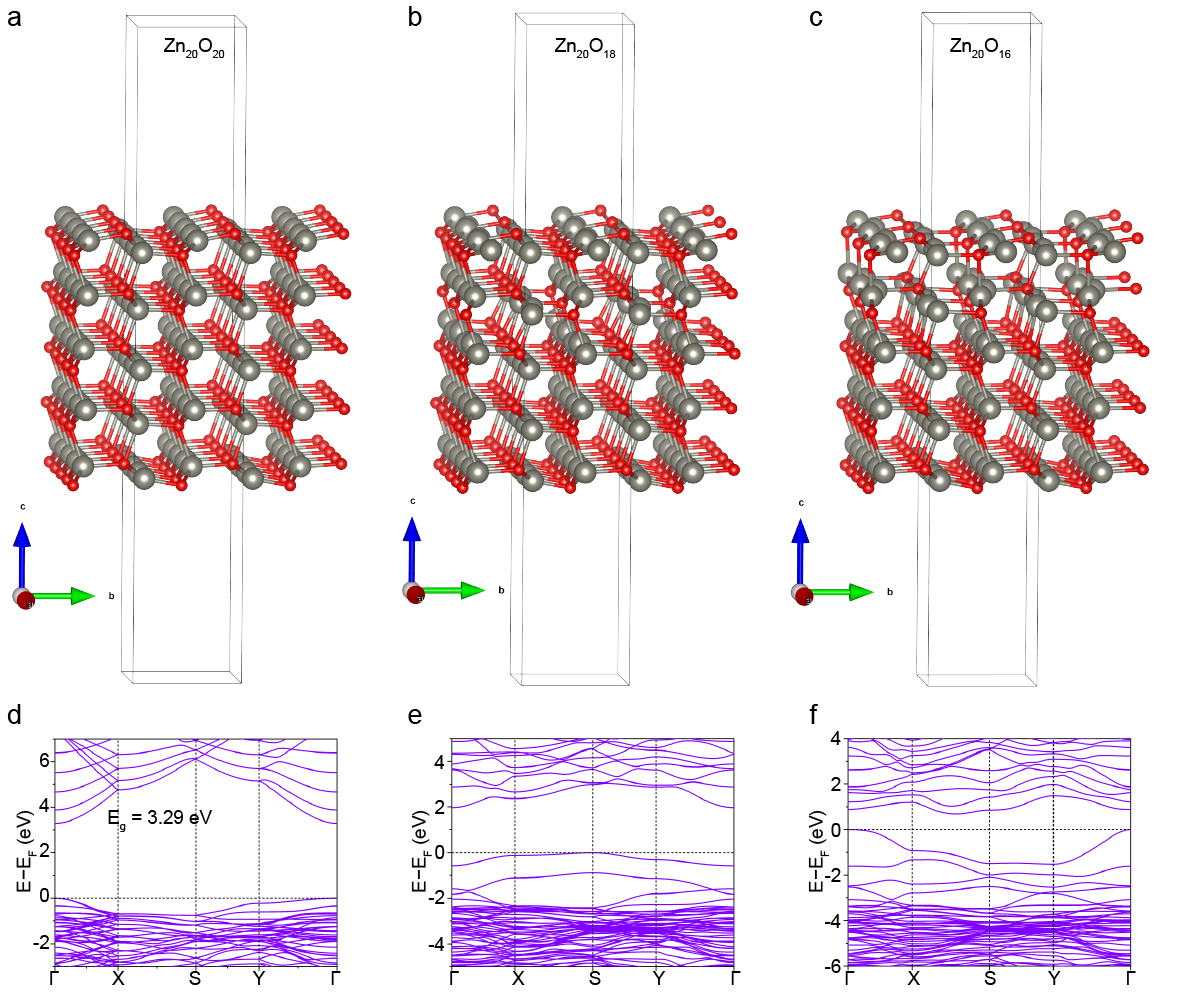


**Figure S10.** **Theoretical calculations of ZnO crystals with and without oxygen vacancies.** (a-c) Atomic structures of Zn_20_O_20_, Zn_20_O_18_ and Zn_20_O_16_ models. (d-f) Corresponding electronic band structures of three models.


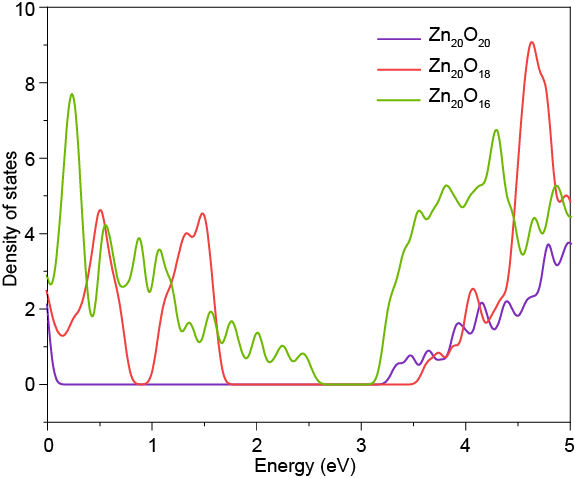


**Figure S11. Calculated density of states (DOS) diagrams of three ZnO crystals with different oxygen vacancy models.**

The X-ray irradiation could ionize water molecules and O_2_ into various hydroxyl radical (OH), cationic hole (H_2_O^+^), O+ 2, O2+ 2and O^+^ ions and radicals ^19-21^. This ionization process remarkably reduces the energetic barrier for eliminating the oxygen vacancies. For verification, density functional theory (DFT) was employed to calculate the energy changes (Fig. S12) associated with this process ^22, 23^. Departing from ZnO crystal with two surface oxygen vacancies (Fig. S12b), we compare the adsorption energies of the system by adsorbing one shows the model of the stoichiometric ZnO surface with two O vacancies. The model after adsorbing oxygen molecule (Fig. S12a) and two oxygen atoms (Fig. S12c). The adsorption energy (*E*_ads_) between ZnO ($10\bar{1}0$) surface with 2*V*_o_ and 2O/O_2_ was defined as following equation:

$$E_{ads}=E_{ZnO(10\bar{1}0) with 2V_{O} +2 O/O_{2}}-E_{\mathrm{ZnO}\left( 10\bar{1}0 \right)with 2Vo}-E_{2O/O_{2}}$$

where $E_{ZnO(10\bar{1}0) with 2V_{O} +2 O/O_{2}}$, $E_{\mathrm{ZnO}\left( 10\bar{1}0 \right) with 2Vo}$ and $E_{2O/O_{2}}$ represent the total energies of the ZnO ($10\bar{1}0$) surface with two O vacancies after adsorbing 2O or O_2_, ZnO ($10\bar{1}0$) surface with 2*V*_o_, and 2O or O_2_, respectively. The minimum energy of O_2_ dissociation at the O vacancy is less than 1 eV ^23^. The adsorption energy between ZnO ($10\bar{1}0$) surface with 2O is -16.48 eV while the adsorption energy (*E*_ads_) between ZnO ($10\bar{1}0$) surface with O_2_ is -5.85 eV. The lower adsorption energy in the former suggests that the system adsorbing two oxygen atoms is more favorable. In another word, the energy barriers for eliminating oxygen vacancies become lower, if the oxygen molecule could be transformed into two oxygen atoms via various experimental tools, such as mild X-ray irradiation. This is the advantage of X-ray irradiation in reducing the surface oxygen vacancies.


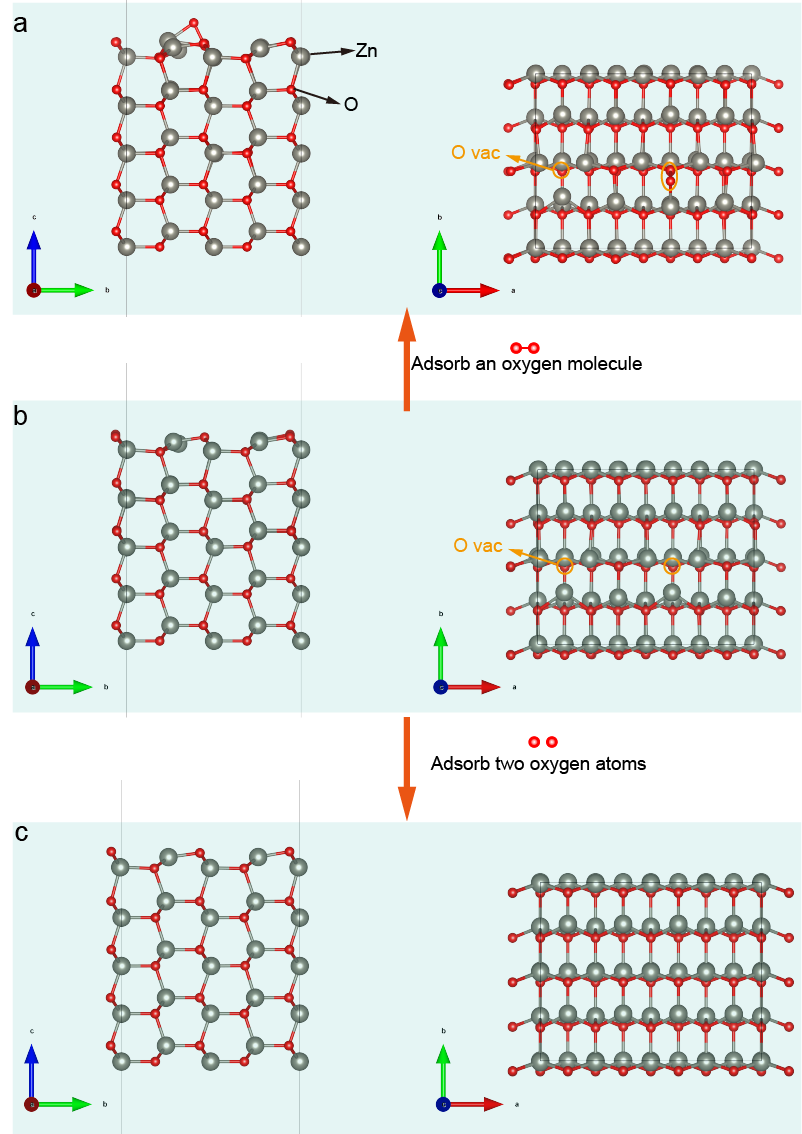


**Figure S12. Calculating the adsorption energy differences between oxygen molecules and two oxygen atoms.** The top- and side-views of ZnO ($10\bar{1}0$) surfaces with two O vacancies and absorbed an oxygen molecule (a), with two O vacancies (b), with two O vacancies and absorbed two O atoms (c), respectively.

# S12. TEM images and profiles of Ga-doped ZnO microwires at three irradiation doses.


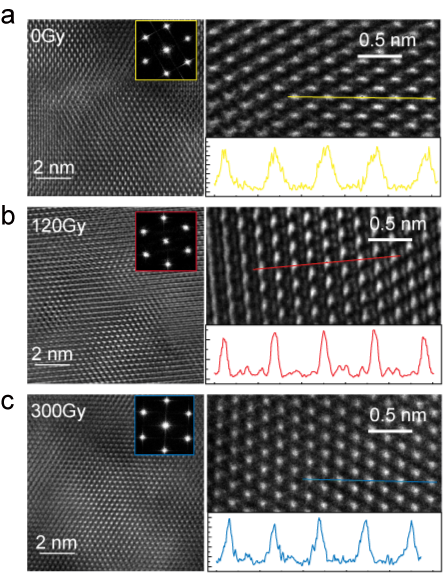


**Figure S13.** **TEM images and corresponding profiles of Ga-doped ZnO microwires after 0 Gy (a), 120 Gy (b) and 300 Gy (c) doses.** The inset FFT images show the lattice structures while the zoom-in TEM images (right, upper) exhibit well-order atoms, re-evidenced by below line profiles. It is concluded that the position and brightness of crystal lattices exhibit better homogeneity after 120 Gy X-ray irradiation.

# S13. Determining the lattice structure from One representative FFT image

**
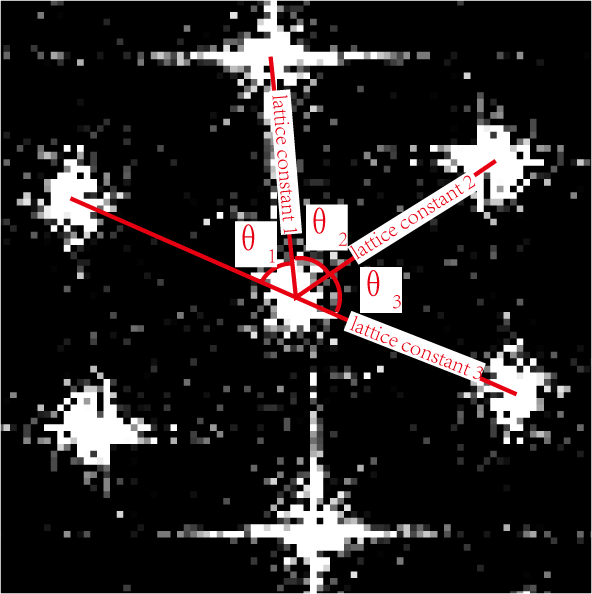
**

**Figure S14. Determining the lattice structure from One representative FFT image.**

The angles and lattice constants are defined in the figure above. The measured angles and lattice constants are shown in the table below.

**Table S6.** **Lattice angles and constants of ZnO microwire at different doses.**

| Dose (Gy) | *θ*_1_ (°) | lattice  constant 1 (nm) | *θ*_2_ (°) | lattice  constant 2 (nm) | *θ*_3_ (°) | lattice  constant 3 (nm) |
| --- | --- | --- | --- | --- | --- | --- |
| 0 | 60.908 | 0.281 | 60.753 | 0.273 | 60.466 | 0.269 |
| 120 | 60.803 | 0.281 | 60.374 | 0.275 | 59.701 | 0.280 |
| 300 | 61.265 | 0.283 | 59.976 | 0.274 | 58.485 | 0.278 |

Table S6 shows the lattice angles and constants at different irradiation doses. Data are derived from measurements of FFT chats in Figure 2 (d)-(f).

**Table S7.** **Lattice angles and constants at different depths under 120 Gy irradiation**

| Distance (μm) | *θ*_1_ (°) | lattice constant 1 (nm) | *θ*_2_ (°) | lattice constant 2 (nm) | *θ*_3_ (°) | lattice constant 3 (nm) |
| --- | --- | --- | --- | --- | --- | --- |
| 0.0 (surface region) | 60.901 | 0.280 | 60.412 | 0.275 | 59.485 | 0.277 |
| 0.5 | 60.442 | 0.276 | 59.862 | 0.274 | 60.544 | 0.279 |
| 2.0 | 61.318 | 0.273 | 60.715 | 0.271 | 59.155 | 0.279 |
| 4.0 | 61.17 | 0.285 | 61.18 | 0.265 | 58.060 | 0.277 |

Table S7 shows the lattice angles and constants at different depths under 120 Gy irradiation. Data are derived from measurements of FFT chats in Figure 4 (c)-(f).

# S14. Normalized time-resolved photoluminescence (TRPL) spectra.


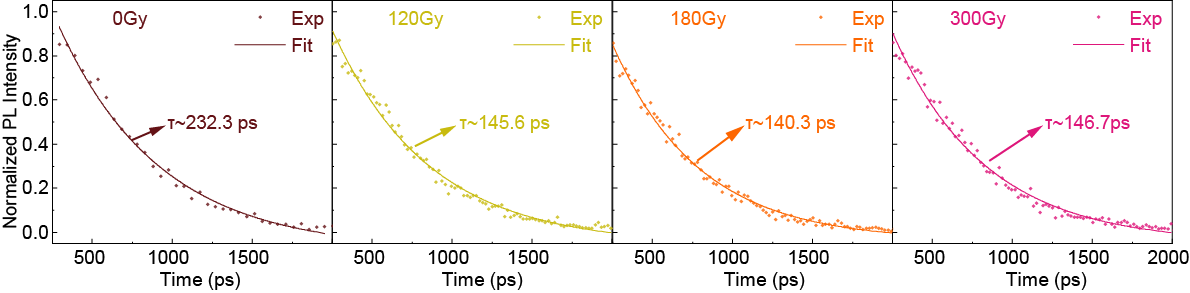


**Figure S15. Normalized TRPL spectra and fitting curves of Ga-doped ZnO microwires at varied irradiation doses.**

To capture the changes of radiation relaxation rates, we performed the optical time-resolved photoluminescence (TRPL) measurements using a steady-state transient fluorescence spectrometer (FLS1000 Edinburgh Instruments at 320 nm). As shown in Figure S15, the decay time of pristine Ga-doped ZnO microwire was fitted to be approximately 232.3 ps, comparable to the values in previous studies ^24, 25^. The PL lifetime first decreases and then increases, the same tendency of PL intensity as function of irradiation doses. These results demonstrate that mid X-ray irradiation can accelerate the radiative recombination rate of electron–hole pairs, resulting in enhanced PL emission.

# S15. Raman signals of pristine and irradiated Ga-doped ZnO microwires


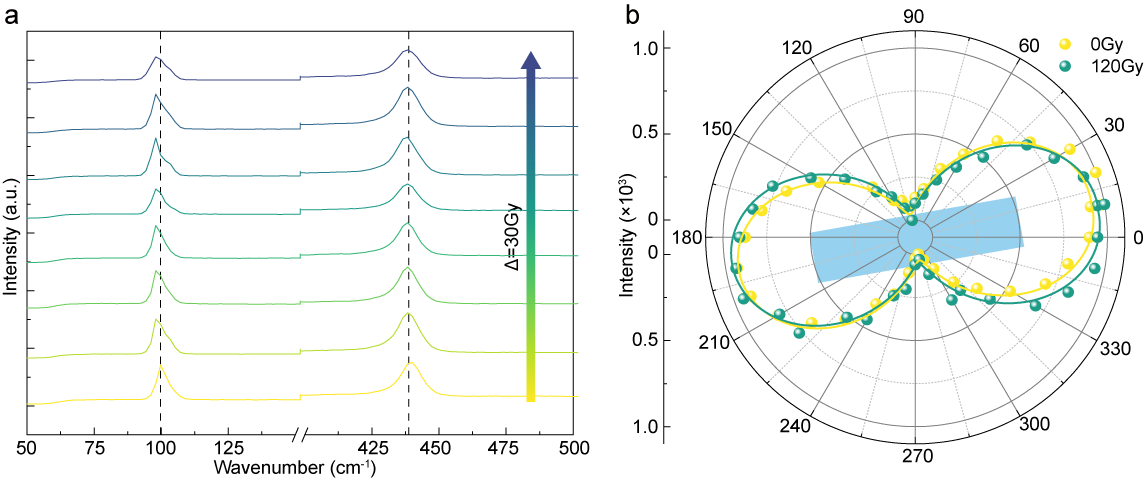


**Figure S16. Raman characteristics of Ga-doped ZnO microwires as function of X-ray irradiations.** (a) Raman spectrum of microwire samples at different radiation doses. The two robust Raman peaks centering at 97.19 cm^-1^ and 435.83 cm^-1^ correspond to $E_{2}^{low}$and $E_{2}^{high}$ modes of ZnO crystal ^26^. The positions of two peaks remain unchanged during dose-varying X-ray irradiations, suggesting the noninvasive nature of X-ray irradiations upon Ga-doped ZnO microwires. (b) The polar plots of angle-resolved Raman intensity of Ga-doped ZnO microwires under 0 Gy and 120 Gy irradiations. As can be seen, the X-ray irradiation barely affect the polarization of ZnO microwires.

# S16. XRD of irradiated Ga-doped ZnO microwires.


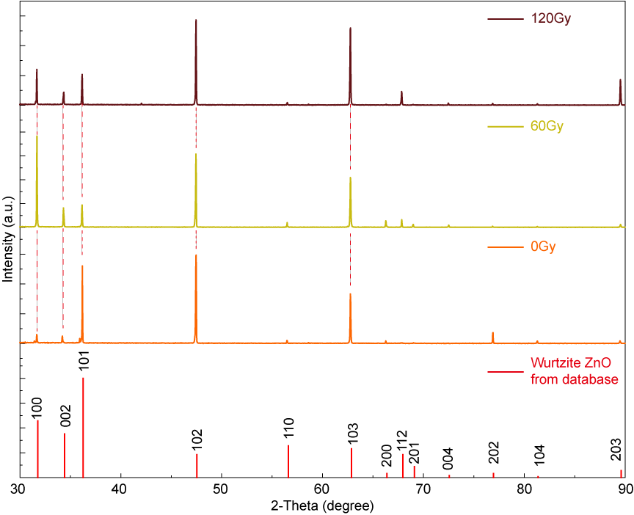


**Figure S17. X-ray diffraction (XRD) of irradiated Ga-doped ZnO microwires at 0 Gy, 60 Gy and 120 Gy.** The pristine and irradiated ZnO shows wurtzite structure with XRD peaks along (100), (002), (101), (102) and (103) planes. No obvious XRD variation was observed after moderate X-ray irradiations, suggesting the superiority of X-ray irradiation approach over other reported methods in giant PL enhancement.

# S17. Description of Ga-doping concentration


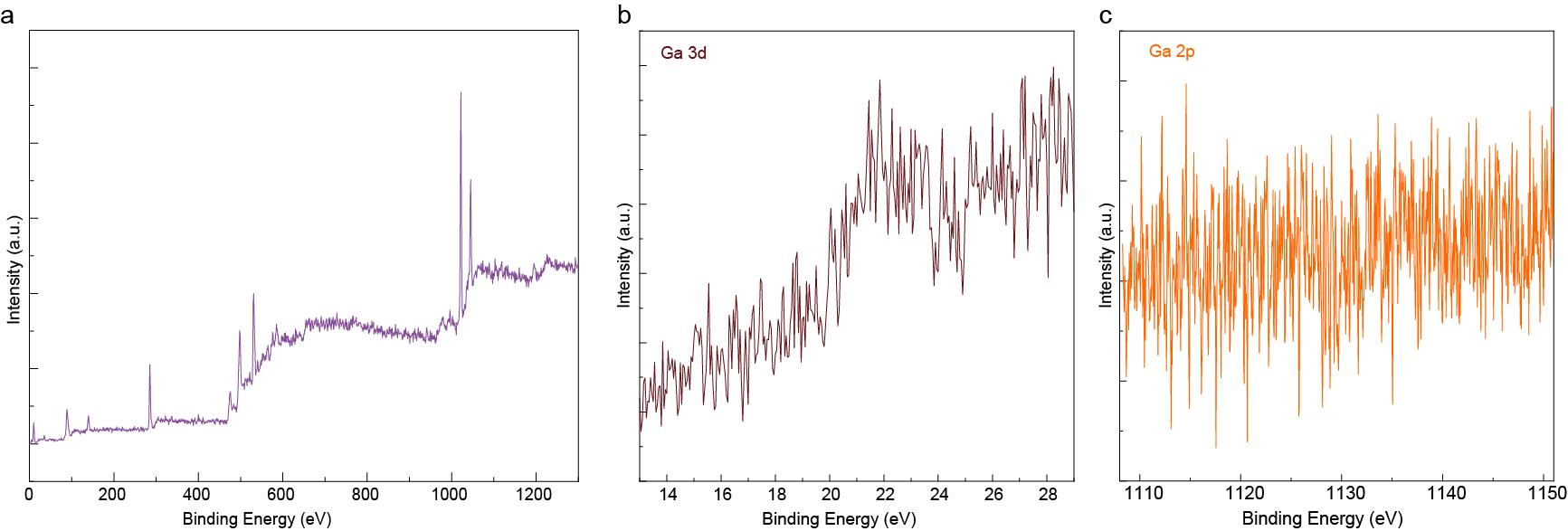


**Figure S18. XPS spectra of Ga-doped ZnO microwire.** (a) survey wide-scan spectra, (b) Ga 3d spectra, (c) Ga 2p spectra.

In our work, the doping concentration of Ga can be qualitatively determined by adjusting the proportion of Ga_2_O_3_ in the precursor during the growth of Ga-doped ZnO on CVD, which cannot be accurately quantified. We detected the Ga-related energy spectrum in the XPS test, as is shown in the following figure. But because the doping content of Ga is less than 1%, Ga-related signal is too weak to be detected in the energy spectrum.


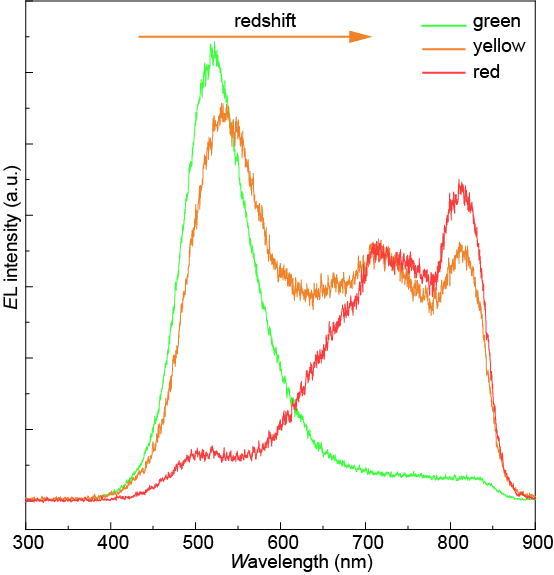


**Figure S19. Electroluminescence spectra of ZnO microwire with different Ga-doping contents.**

Although the concentration of Ga dopant remains less than 1%, the PL and EL performance of Ga-doped microwires significantly varies, which reversely reflects the necessity of Ga dopant and relative Ga concentrations at below 1% region. Figure S19 shows the electroluminescence performances of Ga-doped ZnO microwires with three Ga-doping concentrations (by different loadings of Ga_2_O_3_ precursor). The EL centers redshifts with the increment of Ga dopants evidences the key role of Ga dopant in optical and optoelectrical properties of ZnO nanostructure, but only in a qualitative way.

# S18. Comparison of different methods

**Table S8. Comparison of different methods.**

|  | material | experimental methods | waveband | PL  enhancement | remarks | ref |
| --- | --- | --- | --- | --- | --- | --- |
| 1 | ZnO nanorods | Chemical passivation^127^ | Near band edge emission | 2 times | Cause surface modification | Phys. E: Low-Dimens. Syst. Nanostructures. 2018, 103, 329-337 |
| 2 | ZnO nanoparticles/thin films | Doping (Mg^28^/Al^29^) | Near band edge emission | 2 times | Morphology changes | J. Lumin.  2015, 161, 275-280;  Superlattices and Microstruct.  2021, 151, 106790 |
| 3 | ZnO thin films | Doping (Ga)^3^ | Near band edge emission | 8 times | Surface covered with particles | Phys. Chem. Chem. Phys.,  2019, 21, 15019 |
| 4 | Ga-doped ZnO thin films | Ag ions irradiation  (MeV)^3^ | green emission | 5 time | Need high energy up to MeV | Phys. Chem. Chem. Phys.,  2019, 21, 15019 |
| 5 | ZnO thin films | Ni ions and Au ions irradiations (50 MeV Ni and 120 MeV Au ions)^30^ | visible emission band. | 6 times | Need high energy up to MeV | Radiat. Phys. Chem, 2021, 183, 109400 |
| 6 | ZnO nanorods array | Ga+ ion beam irradiations (5 KeV)^31, 32^ | Near band edge emission | 30 times | Morphology changes; Ga+ions implant into the nanorods, resulting compressive strain | Nano Res. 2015, 8(6), 1857-1864 |
| 7 | Boron doped ZnO films | Br^+6^ ions (80MeV)^32, 33^ | Near band edge emission | 4 times | Need higher energy up to MeV | J. Alloys Compd. 2014, 594, 32-38. |
| 8 | ZnO nanowires | Ar Plasma irradiation (200 W Ar treatment)^33^ | Near band edge emission | 60 times | Morphology changes and slight damage | Nanoscale Res. Lett.  2019, 14, 312 |
| 9 | ZnO/ZnO:Mg shells nanorods | UV laser^34^ | Near band edge emission | 38 times | Unstable; Morphology changes | J. Lumin.  2012, 132, 1885-1889 |
| 10 | single Ga-doped ZnO microwire | X-ray irradiation (160 KeV) | Near band edge emission | 9 times | Negligible morphology changes;  Long-term stability | Our work |

# References

(1) Zhao, C. X.; Li, Y. F.; Chen, Y. C.; et al. Microstructure change of ZnO nanowire induced by energetic x-ray radiation and its effect on the field emission properties. *Nanotechnology* **2013**, *24*, 275703.

(2) Gallino, F.; Pacchioni, G.; Di Valentin, C. J. T. J. o. c. p. Transition levels of defect centers in ZnO by hybrid functionals and localized basis set approach. *J. Chem. Phys* **2010**, *133*, 144512.

(3) Gupta, H.; Singh, J.; Dutt, R.; et al. Defect-induced photoluminescence from gallium-doped zinc oxide thin films: influence of doping and energetic ion irradiation. *Phys. Chem. Chem. Phys* **2019**, *21*, 15019-15029.

(4) Jiang, M. M.; He, G. H.; Chen, H. Y.; et al. Wavelength-Tunable Electroluminescent Light Sources from Individual Ga-Doped ZnO Microwires. *Small* **2017**, *13*, 1604034.

(5) Bekermann, D.; Gasparotto, A.; Barreca, D.; et al. Highly Oriented ZnO Nanorod Arrays by a Novel Plasma Chemical Vapor Deposition Process. *Cryst. Growth Des.* **2010**, *10*, 2011-2018.

(6) Xu, X.; Chen, D.; Yi, Z.; et al. Antimicrobial Mechanism Based on H2O2 Generation at Oxygen Vacancies in ZnO Crystals. *Langmuir* **2013**, *29*, 5573-5580.

(7) Chen, S. Q.; Carraro, G.; Barreca, D.; et al. Aerosol assisted chemical vapour deposition of Ga-doped ZnO films for energy efficient glazing: effects of doping concentration on the film growth behaviour and opto-electronic properties. *J.Mater.Chem.A* **2015**, *3*, 13039-13049.

(8) Frankcombe, T. J.; Liu, Y. J. C. o. M. Interpretation of oxygen 1s X-ray photoelectron spectroscopy of ZnO. *Chem. Mater.* **2023**, *35*, 5468-5474.

(9) Liu, W.; Xu, H.; Ma, J.; et al. Effect of oxygen-related surface adsorption on the efficiency and stability of ZnO nanorod array ultraviolet light-emitting diodes. *Appl. Phys. Lett.* **2012**, *100*, 203101.

(10) Das, D.; Mondal, P. Low temperature grown ZnO:Ga films with predominant c-axis orientation in wurtzite structure demonstrating high conductance, transmittance and photoluminescence. *Rsc Advances* **2016**, *6*, 6144-6153.

(11) Zhang, Q.; Xu, M.; You, B.; et al. Oxygen Vacancy-Mediated ZnO Nanoparticle Photocatalyst for Degradation of Methylene Blue. *Applied Sciences* **2018**, *8*, 353.

(12) Nadupalli, S.; Repp, S.; Weber, S.; et al. About defect phenomena in ZnO nanocrystals. *Nanoscale* **2021**, *13*, 9160-9171.

(13) Giannozzi, P.; Baroni, S.; Bonini, N.; et al. QUANTUM ESPRESSO: a modular and open-source software project for quantum simulations of materials. *J. Phys.: Condens. Matter* **2009**, *21*, 395502.

(14) Giannozzi, P.; Andreussi, O.; Brumme, T.; et al. Advanced capabilities for materials modelling with Quantum ESPRESSO. *J. Phys.: Condens. Matter* **2017**, *29*, 465901.

(15) Perdew, J. P.; Wang, Y. Pair-distribution function and its coupling-constant average for the spin-polarized electron gas. *Phys. Rev. B* **1992**, *46*, 12947-12954.

(16) Perdew, J. P.; Burke, K.; Ernzerhof, M. Generalized Gradient Approximation Made Simple. *Phys. Rev. Lett.* **1996**, *77*, 3865-3868.

(17) Prandini, G.; Marrazzo, A.; Castelli, I. E.; et al. Precision and efficiency in solid-state pseudopotential calculations. *npj Comput. Mater.* **2018**, *4*, 72.

(18) Vrubel, II; Pervishko, A. A.; Yudin, D.; et al. Oxygen vacancy in ZnO-wphase: pseudohybrid Hubbard density functional study. *J Phys Condens Matter* **2020**, *32*, 315503.

(19) Hamill, W. H. Model for the radiolysis of water. *J. Phys. Chem.* **1969**, *73*, 1341-1347.

(20) Sandhu, A. S.; Gagnon, E.; Santra, R.; et al. Observing the Creation of Electronic Feshbach Resonances in Soft X-ray-Induced O_2_ Dissociation. *Science* **2008**, *322*, 1081-1085.

(21) Loh, Z. H.; Doumy, G.; Arnold, C.; et al. Observation of the fastest chemical processes in the radiolysis of water. *Science* **2020**, *367*, 179-182.

(22) Wu, X.; Selloni, A.; Lazzeri, M.; et al. Oxygen vacancy mediated adsorption and reactions of molecular oxygen on the ZnO(101̄0) surface. *Phys. Rev. B* **2003**, *68*, 241402.

(23) Saputro, A. G.; Akbar, F. T.; Setyagar, N. P. P.; et al. Effect of surface defects on the interaction of the oxygen molecule with the ZnO(101̄0) surface. *New J Chem* **2020**, *44*, 7376-7385.

(24) Miao, C. Z.; Xu, H. Y.; Jiang, M. M.; et al. High performance lasing in a single ZnO microwire using Rh nanocubes. *Opt. Express* **2020**, *28*, 20920-20929.

(25) Wan, P.; Jiang, M.; Tang, K.; et al. hot electron injection induced electron–hole plasma lasing in a single microwire covered by large size Ag nanoparticles. *CrystEngComm* **2020**, *22*, 4393-4403.

(26) Shinde, S.; Shinde, P.; Oh, Y.; et al. Structural, optoelectronic, luminescence and thermal properties of Ga-doped zinc oxide thin films. *Appl. Surf. Sci.* **2012**, *258*, 9969-9976.

(27) Ali, A.; Rahman, G.; Ali, T.; et al. Enhanced band edge luminescence of ZnO nanorods after surface passivation with ZnS. *Physica. E. Low. Dimens. Syst. Nanostruct.* **2018**, *103*, 329-337.

(28) Arshad, M.; Ansari, M. M.; Ahmed, A. S.; et al. Band gap engineering and enhanced photoluminescence of Mg doped ZnO nanoparticles synthesized by wet chemical route. *J. Lumin.* **2015**, *161*, 275-280.

(29) Pon, V. D.; Wilson, K. J.; Hariprasad, K.; et al. Enhancement of optoelectronic properties of ZnO thin films by Al doping for photodetector applications. *Superlattices Microstruct.* **2021**, *151*, 106790.

(30) Singh, R.; Gupta, H.; Mehra, R.; et al. Tuning of defects induced visible photoluminescence by swift heavy ion irradiation and thermal annealing in zinc oxide films. *Radiat. Phys. Chem.* **2021**, *183*, 109400.

(31) Yadian, B.; Chen, R.; Liu, H.; et al. Significant enhancement of UV emission in ZnO nanorods subject to Ga^+^ ion beam irradiation. *Nano Research* **2015**, *8*, 1857-1864.

(32) Kumar, V.; Kumar, V.; Som, S.; et al. Role of swift heavy ions irradiation on the emission of boron doped ZnO thin films for near white light application. *J. Alloys Compd.* **2014**, *594*, 32-38.

(33) Li, H.; Tang, J.; Lin, F.; et al. Improved optical property and lasing of ZnO nanowires by Ar plasma treatment. *Nanoscale Res. Lett.* **2019**, *14*, 1-8.

(34) Wang, Y. H.; Duan, W. J.; Wu, Z. L.; et al. Enormous enhancement of ZnO nanorod photoluminescence. *J. Lumin.* **2012**, *132*, 1885-1889.
